# Mechanical Characterization and Structural Analysis of Latex-Containing and Latex-Free Intermaxillary Orthodontic Elastics

**DOI:** 10.3390/polym14214488

**Published:** 2022-10-23

**Authors:** Zsuzsanna Gurdán, Kinga Turzó, Laura Lőrinc, Péter Szabó, Kristóf Karádi, András Lukács, Roland Told, Kinga Kardos, Péter Maróti

**Affiliations:** 1 Department of Paediatric and Adolescent Dentistry, Clinical Center, Medical School, University of Pécs, Tüzér Str. 1., H-7623 Pécs, Hungary; 2 Environmental Analytical and Geoanalytical Research Group, University of Pécs, Ifjúság Str. 20, H-7624 Pécs, Hungary; 3 Department of Biophysics, Clinical Center, Medical School, University of Pécs, Szigeti Str. 12, H-7624 Pécs, Hungary; 4 3D Printing and Visualisation Centre, University of Pécs, Boszorkány Str. 2, H-7624 Pécs, Hungary; 5 Medical Simulation Education Centre, Medical School, University of Pécs, Szigeti Str. 12, H-7624 Pecs, Hungary

**Keywords:** intermaxillary elastics, orthodontics, force degradation, material testing, tensile test, cyclical fatigue test, scanning electron microscopy

## Abstract

Class II malocclusion is one of the most common dental anomalies and the use of intermaxillary elastomers is the standard method in its treatment. However, orthodontic elastics cannot exert continuous force over a period of time due to force degradation. Our goal was to mechanically characterize the different types of elastomers during static and cyclic loads, based on uniform methodology and examine the morphological changes after loading. Ten types of latex-containing and four latex-free intermaxillary elastics were examined from six different manufacturers. To determine the mechanical characteristics of the elastomers, tensile tests, cyclical tensile fatigue tests and 24 h relaxation tests were performed, and the elastics were also subjected to scanning electron microscopy (SEM) and Raman spectroscopy. Regardless of the manufacturer, the latex-containing elastomers did not show significant differences in the percentage of elongation at break during the tensile test. Only one type of latex-containing elastomer did not tear during the 24 h cyclical fatigue test. Fatigue was confirmed by electron microscopy images, and the pulling force reduced significantly. During the force relaxation test, only one latex-free ligature was torn; the force degradation was between 7.8% and 20.3% for latex ligatures and between 29.6% and 40.1% for latex-free elastomers. The results showed that dynamic loading was more damaging to ligatures than static loading, latex-containing elastomers were more resistant than latex-free elastics, and which observation could have clinical consequences or a potential effect on patient outcome.

## 1. Introduction

Class II malocclusion is a serious reason why patients look for orthodontic treatment [1,2,3,4,5,6]. Many orthodontic treatments have been invented and developed for treating malocclusion according to the age and the dentoskeletal characteristics of patients, oral hygiene and the patient’s will to follow the therapy in a given period of time. Patients with skeletal malocclusion may suffer from dental deformities, bruxism, teeth crowding, trismus, mastication difficulties, breathing obstruction and digestion disturbance, if the pathology is not corrected in time [7].

A common procedure in the treatment of class II malocclusions in a growing of patients is a two-step approach: functional therapy during the growth period and tooth arch harmonization with fixed appliances, respectively. The use of intermaxillary elastics has been a standard method in the improvement of class II malocclusions since the early days of orthodontic maintenance; their use was pioneered by Calvin S. Case of Chicago and Henry A. Baker of Boston in the 1890s [8,9,10].

Intermaxillary elastics are made of either latex (natural rubber) or synthetic polymer materials. Latex elastics are more widely used since they present improved properties, such as greater flexibility, lower costs and a greater capacity for returning to the original shape and form after undergoing deformation. However, allergies caused by latex protein, such as swelling, stomatitis, erythematous changes in the oral cavity, and more severe respiratory and anaphylactic reactions, occur in 3–17% of cases [11].

The production of elastomers includes a dipping process on a steel mandrel of varying thickness, and then the rubbers are sliced; the more dips, the thicker the resulting tube. Based on the thickness, which plays a role in the characteristics and quality of the pipe, manufacturers use the term “light” or “heavy” elastics. In addition, three other factors are important: the lumen of the pipe, the width of the cut and the properties of the elastic material [12].

Orthodontic elastomers cannot exert continuous force. The decrease of the elastic force occurs during the elongation of the molecular chains, during which the rounding and elongation of the polymer chains can be observed. Furthermore, if large, excessive forces are used, the chains may slip on each other, resulting in the permanent deformation of the material [13].

Vieira et al. compared the strength degradation of latex and synthetic intermaxillary elastics of the same product (Dental Morelli). Based on in vitro test results, latex elastics have better mechanical properties [14]. In a 24 h in vitro study, Klabunde et al. evaluated force degradation after the dynamic loading of American Orthodontics-type elastics. Latex elastics showed significantly greater force reduction, so the authors recommend more frequent replacement during treatment [15].

Some researchers recommend daily replacement of intermaxillary elastics, while others recommend changes every 4 h [16], or after 8 h, of wear, so that the application of new elastomers is considered optimal [17]. This is explained by the rapid decrease in force observed in a dynamic environment, which can be observed after only a few hours. According to other authors, intermaxillary elastics can be worn for several days because there is no difference in the 24 h and 72 h force degradation values [18]. The elastomers must be removed for the patient to perform oral hygiene and during the consumption of meals. Both in vivo and in vitro studies confirmed that comparison of the loss of elasticity strength at intervals of 24 and 48 h showed no significant difference. Thus, these studies also recommend replacing elastomers every 24 h [14].

Interestingly, the value of pH has no significant effect on force degradation, as it was observed in previous studies [19,20].

In orthodontic practice, the use of elastics has some advantages, such as biocompatibility, low costs, easy installation and removal by the patient. In order to select the most appropriate device for a given tooth movement, clinicians need to be aware of the force exerted by flexibility and how that force decreases over time. While a large number of studies have been conducted on latex elastic materials, the number of studies on non-latex elastic materials is small and controversial [21,22]. According to some authors, the properties of non-latex elastic materials would need to be greatly improved before they can be considered as an acceptable alternative to latex elastic materials [23]. It would also be interesting to see if the latest available latex elastic materials have better mechanical properties compared to those tested 10 or more years ago. As a result of such improvements, updated clinical recommendations for the use of orthodontic flexibility may emerge [24].

In previous studies, there were big differences between the methodology of the studies analyzed, which makes it difficult to draw the right conclusions. At the beginning of our study, we assumed that there are significant differences in the elongation at the break of tensile between the products of different manufactures. Therefore, the aims of the present study are to test different types of intermaxillary elastics in terms of mechanical behavior, under standardized testing conditions, and evaluate the force degradation by 24 h cyclic tensile fatigue test and tension relaxation test. We hypothesize that there is a significant difference between latex-containing products and latex-free elastics during dynamic testing. The study also examines the morphological changes of intermaxillary elastics after loading with scanning electron microscopy and determines the chemical composition of the elastomers based on the peak absorption intensities, using Raman spectroscopy [25,26]. According to the literature review, these parameters have not been investigated in a large number of studies, despite the fact that they have significant clinical relevance. Additionally, the study provides recommendations to orthodontists for the treatment of various class II anomalies with different elastomers.

## 2. Materials and Methods

### 2.1. Structure of the Study

**Figure 1:**
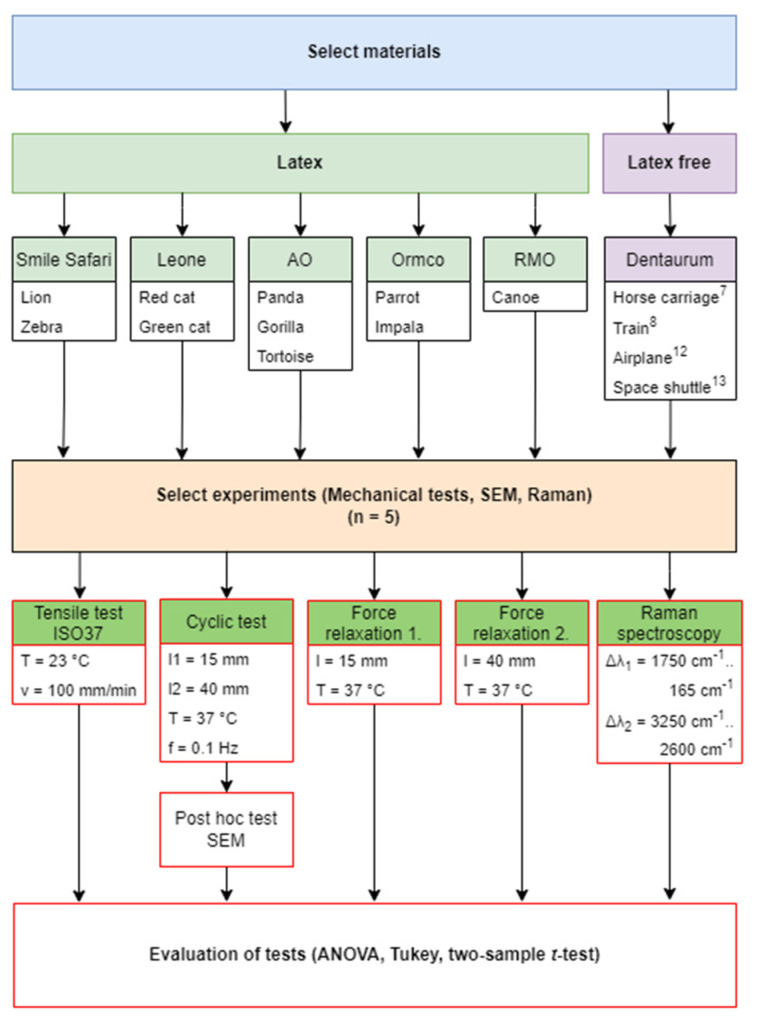
The protocol of the study. ^7,8,12,13^ The name of the elastomers.

The aim of the present study was to critically evaluate the change in orthodontic ligatures in terms of static and cyclic forces. The protocol diagram of the study is shown in Figure 1.

### 2.2. Materials and Specimens

The following ligature manufacturers and products were selected for the tests.

- Smile safari (Ortho Organizers, Inc., Carlsbad, CA, USA).
- Leone (Leone S.p.A., Firenze, Italy).
- AO (American Orthodontics, Sheboygan, WI, USA).
- Ormco (Ormco Corporation, Glendora, CA, USA).
- Dentaurum (75228 Ispringen, Germany).
- RMO (Rocky Mountain Orthodontics, Denver, CO, USA).

In this study, 14 types of intermaxillary elastomers were investigated, the ligatures are described in detail in Table 1. For all measurement, 5-5 pieces were tested for each type of elastomer. A total of 350 elastomers were examined.

### 2.3. Material Testing

#### 2.3.1. Tensile Test

Tensile testing of elastic ligatures was performed by a Zwick/Roell Z5.0 biaxial universal testing machine (ZwickRoell, 89079 Ulm, Germany) at room temperature (23 °C ± 1 °C), using 5-5 pieces of each specimen. Tests were conducted according to the ISO 37:2011 guideline [27]. Ligatures were tested as a ring shape. The ligatures were fixed to the grips with steel hooks with a thickness of 1.4 mm. The crosshead speed was set to 100 mm/min.

#### 2.3.2. Cyclical Tensile Fatigue Test

For the cyclic tensile fatigue test, a thermostatic chamber was built to maintain the ambient temperature at 37 °C ± 1 °C during the tests in order to mimic the environment of oral cavity. Heating was performed using a Julabo ED thermostat (JULABO GmbH, Seelbach, Germany), which circulated the 37 °C water in a copper spiral in the chamber.

**Figure 2:**
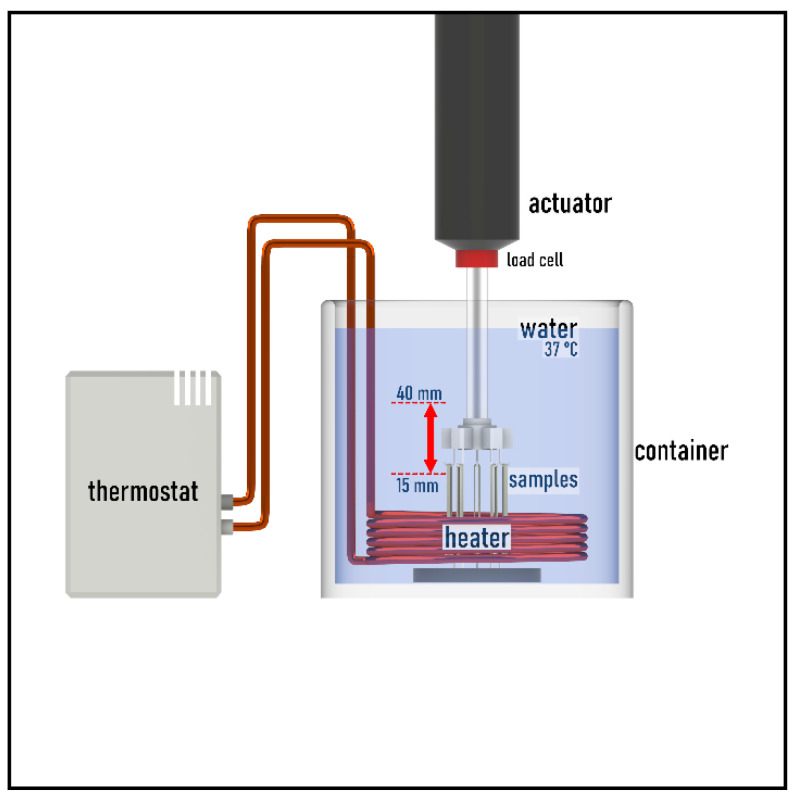
The temperature chamber and the measuring system.

The chamber was added to a Zwick/Roell e/m actuator (ZwickRoell, Ulm, Germany). A 5 kN load cell was placed on the testing machine, which was calibrated with a PCE-BS 3000 laboratory scale (PCE Holding GmbH, Hamburg, Germany) before the test. The sample holder of the chamber was designed to keep measuring five ligatures at once, which resulted in more efficient use and faster measurements (Figure 2). The test lasted for 24 h and during this time the holder stretched the ligatures between 15 mm and 40 mm with sinusoidal movement on 0.1 Hz frequency.

**Figure 3:**
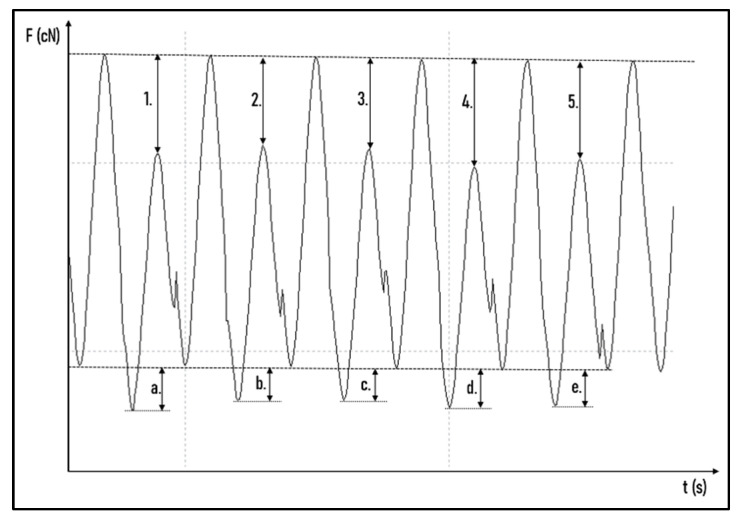
Determination of individual tensile forces. The tensile force of the first (1.), the second (2.), the third (3.), the fourth (4.), and the fifth (5.) ligature at 40 mm and the tensile force of the first (a.), the second (b.), the third (c.), the fourth (d.), and the fifth (e.) ligature at 15 mm.

The force degradation curve was determined using the OriginPro 2018 program (OriginLab Corporation, Northampton, MA, USA) with maximum peak detections when stretched at 40 mm and minimum peak detections when stretched at 15 mm. To determine measurement uncertainties during the 24 h test, the ligatures were unhooked one by one from the load cell at 0 h, 1 h, 3 h, 6 h, 12 h and 24 h for one full sinusoid cycle (Figure 3). Following this step, it was possible to calculate the ligatures’ individual values from the force decrease. The individual values were summed and then compared with the load cell value. If the two values were different, each value was corrected proportionally so that the summed value was equal to the load cell value (Supplementary Materials: Measured values and Statistic.xlsx and cyclic fatigue individual value sheet).

#### 2.3.3. 24 h Tension Relaxation

**Figure 4:**
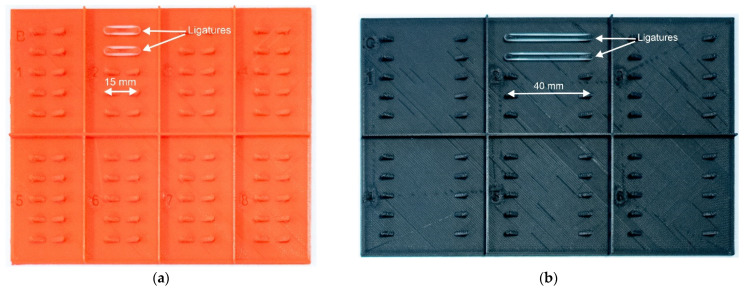
(**a**) The 15 mm ligature holder with two sample ligatures. (**b**) The 40 mm ligature holder with two sample ligatures.

Each type of ligature was stretched to a constant length of 15 mm and 40 mm for 24 h on a 3D printed holder. In each case, the number of specimens tested was five. During the 24 h, the samples were stretched with a sample holder (Figure 4), and the tensile forces of the ligatures were measured at 0 h, 12 h and 24 h. The test machine was the same Zwick/Roell e/m actuator, which was used in the cyclical tests. The ligatures were kept in 37 °C distilled water in the holder and also during the measurements, in order to mimic the oral cavity.

#### 2.3.4. Scanning Electron Microscopy

After the cyclic fatigue tests, SEM imaging was carried out on the samples with 100× and 1000× magnification using a JEOL JSM-IT500HR device (JEOL Ltd., Akishima, Tokyo, Japan). Before imaging, the samples were coated with gold using a JEOL JFC-1300 auto-fine coater. The surface images were captured using the secondary electron detector, and the accelerating voltage was set to 5 kV. The working distance was around 13 to 15 mm, depending on the height of the samples.

#### 2.3.5. Raman Spectroscopy Measurements

Raman scattering is a powerful spectroscopical method widely used in chemistry and biology, but also in archeology as it is very sensitive to the chemical composition of the studied material. Raman spectra were measured with a Thermo Scientific DXR Raman Microscope (Thermo Fisher Scientific, Waltham, MA, USA). The rubber samples were placed on the slide and were first observed with a camera. For data collection, the research group used a 780 nm laser at 10 mW laser power and collected data with a 10× camera lens. The focus was optimized in order to provide the clearest spectrum with the highest intensity (CPS). Typically, 5 spectra, for a 2 s exposure each, were averaged for the measurements. The instrument covered the 400 cm^−1^–4000 cm^−1^ spectral range with a resolution ~4 cm^−1^.

#### 2.3.6. Statistical Analysis

In case of cyclic fatigue tests and force relaxation, the individual forces measured on the same type of ligatures at different time points were compared using one-way ANOVA. The value sets for the same time points were compared pairwise using a Tukey post hoc test. The confidence interval was 95%. The cyclic and static fatigue tests results were compared by a two-sample *t*-test on the same products and with the same time points. The confidence interval was 95%. The statistics and curve draw were made with the OriginPro 2018 program (OriginLab Corporation, Northampton, MA, USA).

## 3. Results

### 3.1. Tensile Test

**Figure 5:**
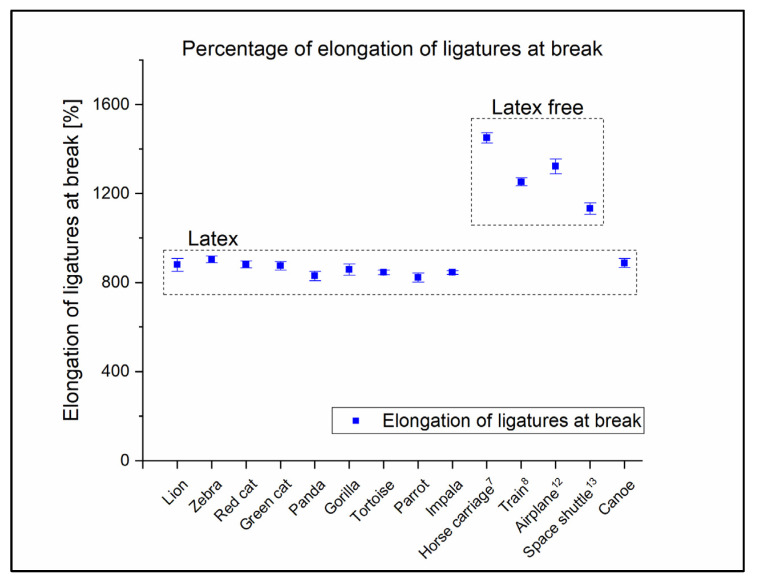
Tensile test elongations in %. Results clearly show that the elongations at break of the latex ligatures are approximately equal regardless of manufacturer.

Considering the dental industry applications of these ligatures, tensile strength (*F*_max_) was measured in cN instead of MPa. Additionally, elongation measured in mm is more important than the elongation percentage, with regard to the dental industry (Table 2). Nonetheless, the standard elongation percentages are also indicated in Table 2, thereby helping the comparison of the raw materials. These results show that the latex ligatures broke, approximately, at the same elongation regardless of the manufacturer (Figure 5).

### 3.2. Cyclical Tensile Fatigue Test and Stress Relaxation

The results of the cyclical and stress relaxation tests were also compared to each other, and the results are therefore evaluated in the same subchapter.

**Figure 6:**
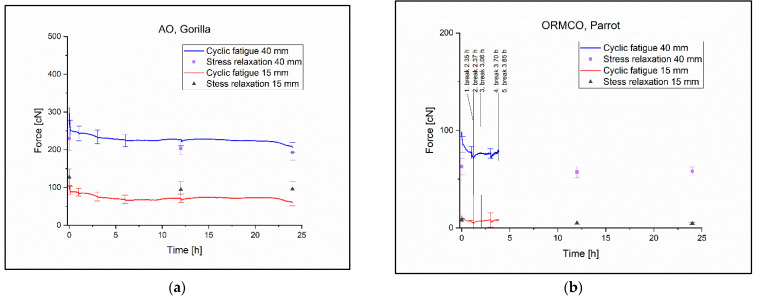
(**a**) The Gorilla ligature cyclic and relaxation diagram. (**b**) The Parrot ligature cyclic and relaxation diagram with the times of the torn specimens.

During the 24 h of cyclical tests, the Gorilla type containing latex was the only one that did not present any torn ligatures (Figure 6a). Significant changes were observed in the pulling force between 0 h and 24 h: 255.5 cN ± 20.68 cN at 0 h and 208.3 ± 13.38 cN at 24 h (*p* = 0.003). The same observations were made considering the pulling force at 40 mm elongation in the Tukey test at 1 h and 24 h: 244.2 cN ± 19.28 cN was measured at the 1 h (*p* = 0.036) (Table 3). The cases in which changes were less than 5% in the confidence interval with the Gorilla ligature at 40 mm elongation were: between 3 h and 6 h, where the pulling force was 233.3 cN ± 18.06 cN at 3 h and 225.2 cN ± 16.13 cN at 6 h, between 3 h and 12 h, where the pulling force was 225.6 cN ± 16.58 cN at 12 h, and between 6 h and 12 h. The *p* values and the changes of the averages are shown in Table 3. The changes were less than 5% in the confidence interval at 15 mm elongation in the following cases: between 0 h and 1 h, between 3 h and 6 h, between 3 h and 12 h, and between 6 h and 12 h. All cyclical and stress relaxation test results can be found in the Supplementary Materials: Cyclic tests’ and Stress relaxations’ diagrams folder. Their descriptive statistics can be found inside the Measured values and Statistic.xlsx file in the cyclic descriptive and stress relaxation individual values sheets.

Within the group of the latex-containing ligatures, it was the ‘Parrot’ in which the ligatures tore quickly, with the last ligature at 3.58 h (Figure 6b). The only significant change occurred in the Tukey test at 40 mm elongation between 0 h and 1 h, where the pulling force was 88.8 cN ± 8.11 cN at 0 h and 75.9 ± 6.4 cN at 1 h (*p* = 0.035) during the cyclic measurement (Table 3).

**Figure 7:**
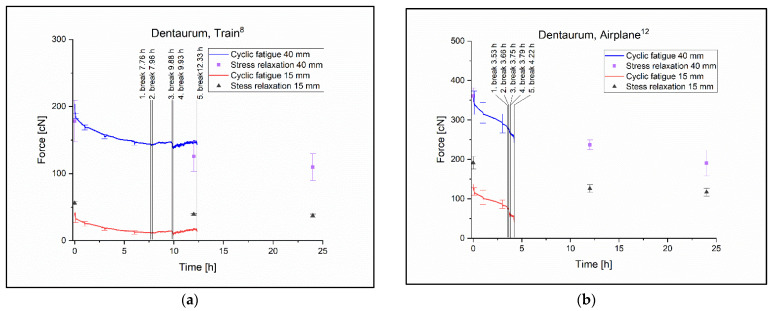
(**a**) The Train^8^ ligature cyclic and relaxation diagram with the times at break. (**b**) The Parrot airplane^12^ cyclic and relaxation diagram with the times at break.

None of the latex-free ligatures withstood the cyclic test for 13 h. With this group, the Train^8^ was the most resistant with the last ligature torn at 12.33 h (Figure 7a). The change in pulling force was significant almost everywhere but this is due to the small standard deviations in the ligatures (Table 4.). The pulling force was 196.5 cN ± 3.9 cN at 0 h and 146.5 cN ± 2.48 cN at 6 h in the Tukey test at 40 mm elongation with a p value of *p* = 0 (Table 4).

The worst performing ligature was the Airplane^12^ with the last ligature torn at 4.22 h and the pulling force decreasing approximately linearly during the test (Figure 7b). The pulling force was 344.7 cN ± 29.68 cN at 0 h and 291.2 cN ± 23.94 cN at 3 h, the *p* value in the Tukey test with 40 mm elongation was *p* = 0.3 (Table 4).

For the cyclic test, the comparison of force degradation was measured by the ANOVA test; the only test specimen that showed no significant difference was the Green Cat ligature. The pulling force at 40 mm elongation was 200.7 cN ± 36.33 cN at 5 min and 189 cN ± 48.00 cN at 24 h (*p* = 0.92). In case of 15 mm elongation, the pulling force was 69.48 cN ± 15.57 cN at 5 min and 63.9 cN ± 23.04 cN at 24 h (*p* = 0.63). Only a single test specimen of the Green Cat ligature was torn during the test.

For the force relaxation test, the comparison of force degradation was measured by the ANOVA test; the only test specimen that showed no significant difference was the Gorilla ligature. The pulling force at 40 mm elongation was 229.8 cN ± 30.62 cN at 5 min and 192.6 cN ± 19.68 cN at 24 h (*p* = 0.11). In case of 15 mm elongation, the pulling force was 127.2 cN ± 22.54 cN at 0 h and 95.8 cN ± 19.18 cN at 24 h (*p* = 0.078). Only a single test specimen of the Green Cat ligature was torn during the test.

All ANOVA and Tukey post hoc test results can be found in the supplementary materials: Measured values and Statistic.xlsx and ANOVA and Tukey sheets.

The force relaxation test was less destructive to the ligatures than the cyclical test. During these tests, the only ligature torn was the Horse Carriage^7^ type at 40 mm elongation.

The results of the cyclic fatigue and force relaxation were compared by a two-sample *t*-test. In the case of 40 mm elongation, three ligatures showed significant differences between the cyclic and force relaxation tests’ values at 0 h on the two-sample *t*-test. These ligatures were Parrot, Space Shuttle^13^ and Canoe (Table 5).

At 12 h, the Canoe, and at 24 h, the Zebra, showed significant differences. In the case of the Green Cat and Impala specimens, the changes were less than 5% in the confidence interval at 0 h and for the Impala ligature also at 24 h.

At 15 mm elongation, all ligatures showed significant differences between the cyclic and force relaxation tests at 0 h, 12 h and 24 h in the two-sample *t*-test, except the Gorilla ligature, for which there was no significant difference in any case (Table 5). It can be observed that significant changes are more frequent at 15 mm elongation.

The percentage of force degradation was also calculated. During the first 12 h of static measurement at 40 mm length, Green Cat showed the most substantial reduction (20.3%) with Canoe producing the least (7.8%) amongst the latex-containing ligatures, while the force degradation was between 29.6% and 40.1% for the latex-free ligatures. When the intermaxillary elastics were stretched to 15 mm length, the reduction in force was between 7.7% and 37.4% in case of the latex-containing ligatures, and 29.6–39.3% for the latex-free ligatures within the first 12 h. Between 12 h and 24 h, the force degradation was lower, and in some cases, a small increase was detected.

All force degradation percentage results can be found in the Supplementary Materials: Measured values and Statistic.xlsx file and the percentage of force degradation sheet.

### 3.3. Scanning Electron Microscopy

**Figure 8:**
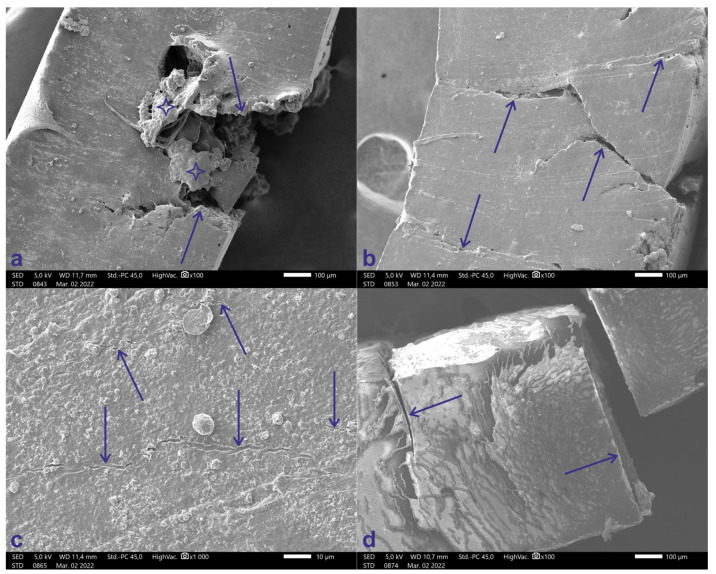
(**a**) SEM record of Lion ligature at 100× magnification. Arrows show the irregular shape of the crack, blue stars indicating the fluffing. (**b**) Red cat SEM recorded at 100× magnification. Arrows show the irregular shape of the crack. (**c**) Gorilla ligature SEM recorded at 1000× magnification. Only small cracks are visible on the surface (blue arrows). (**d**) Horse carriage ligature SEM recorded at 100× magnification. The cracks are rectilinear and perpendicular to the outer surface.

Signs of fatigue can be clearly observed on the latex ligatures, depending on the additives. The typical signs:

Figure 8a shows a Lion ligature SEM recorded at 100× magnification. The arrows indicate the irregular shape of the crack. In some cases, fluffing can be observed next to the hook, likely due to the tearing of the polymer fibers (blue star).

Figure 8b shows a Red cat ligature SEM recorded at 100× magnification. The arrows indicate the irregular shape of the crack.

Figure 8c shows a Gorilla ligature SEM recorded at 1000× magnification. Only small cracks are visible on this ligature’s surface (blue arrows).

Figure 8d shows a Horse carriage^7^ ligature SEM recorded at 100× magnification. The cracks are rectilinear and perpendicular to the outer surface. There are no traces of fatigue on the latex-free ligatures.

The images of all ligatures at 100× magnification can be found in the supplementary materials, inside the SEM folder.

### 3.4. Raman Spectroscopy

The 14 different types of ligatures were also tested using Raman technique and the resulting spectra were then compared. Based on the Raman measurements, the elastomers were divided into two categories, which also corresponded to the observed color of the ligatures. In the latex group were included samples 1–9 and sample 14, the latex-free group consisted of samples 10–13.

**Figure 9:**
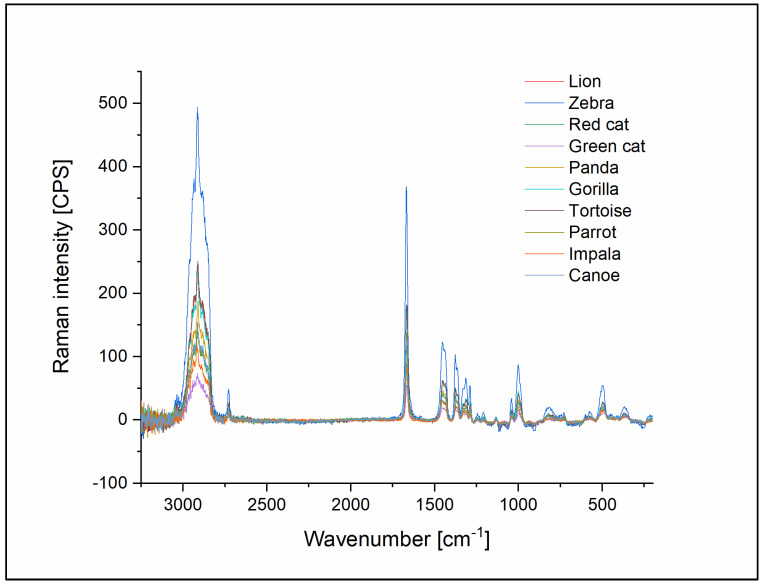
Raman spectra of all latex ligatures with identical peaks show that there is no significant difference between the latex ligatures.

The Raman spectra of the latex ligatures showed typical vibrational features known from the literature. The most salient features were the peak at 1667 cm^−1^ and the peak at 2914 cm^−1^. The peak at 1667 cm^−1^ was assigned earlier [28,29,30] as a C=C stretch. The strong signal at ~2914 cm^−1^ is rather a combination of three poorly resolved peaks, as seen earlier [30]. The two shoulders (2881 cm^−1^ and 2932 cm^−1^) were assigned as asymmetric CH_2_ stretches. The central peak (2914 cm^−1^) was a symmetric stretch of CH_3_. The Raman measurements did not show differences between the latex-based samples, which is not unexpected, as, for example, the increasing silica of natural rubber was hardly observed previously [30]. The lack of additional peaks from the added materials could be a result of the low concentration used or the lack of the Raman activity of the added compound (Figure 9).

**Figure 10:**
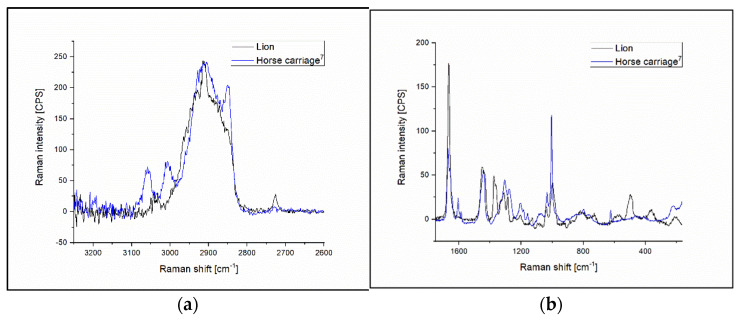
(**a**) Raman spectra of Lion (represents latex ligature group) and Horse carriage^7^ (represents latex-free ligatures) elastomers at 3250 cm^−1^–2600 cm^−1^ region. (**b**) Raman spectra of Lion and Horse carriage^7^ elastomers at the range of 1750 cm^−1^–165 cm^−1^.

Comparing latex-based rubbers to a latex-free (Horse carriage^7^) one led to interesting differences in the obtained Raman spectra. The 367 cm^−1^ and 500 cm^−1^ peaks are missing from the latex-free material (Figure 10b); the later peak was assigned as =CC_2_ rocking and scissoring vibrational mode. Another intense vibrational mode is missing from the latex-free rubber at 1313 cm^−1^, which was assigned as a CH_2_ twisting mode in natural rubber. Examining the spectra recorded at higher frequencies, two additional peaks can be observed at 3006 cm^−1^ and 3058 cm^−1^ in the spectrum of the latex-free rubber (Figure 10a). These differences show that the composition of the latex-free rubbers from a chemical point of view are very similar to the latex-based materials, although significant differences can be observed as well.

## 4. Discussion

Orthodontics is a complex field, and in addition to the professional knowledge of an orthodontist, patient cooperation is essential to achieve a successful outcome. To this end, the primary consideration for corrective devices is to provide fast, efficient treatment in the most comfortable form possible. It is well documented in the literature that intermaxillary elastics are effective in the correction of the anteroposterior relationship of the dentition.

Two main types of orthodontic elastomers are known. Natural latex is an isopropene polymer composed of heavy molecules, proteins and fatty acids. During the manufacturing process, natural latex is mixed with ammonia and stabilizers such as zinc oxide, as well as antioxidants, anti-ozone and other chemicals, which increase the product’s resistance and flexibility, and extend the shelf life of elastics. However, in addition to latex proteins, the added components may also cause allergic reactions [31].

The number of latex sensitivities has recently increased, so the development of alternative products has become a necessity. An example is polyurethane rubber, which is made of synthetic polymers [20,32,33].

In this study, the elongation at break of the tensile strength tests was shown. The results of our experiment did not support our assumption regarding the elongation of the tensile strength, which does not show a significant difference between products made with latex-containing elastomers from different manufacturers. Raman spectroscopy results also showed that there is no difference in the composition of these materials. Interestingly, no such phenomenon was observed with latex-free ligatures, although Raman spectroscopy showed no significant difference between them.

Previous studies have compared the degree of deformation and the degradation of intermaxillary elastics. Latex elastics showed better results than non-latex elastics through these dimensions. In this study, the force degradation of latex ligatures in the first 12 h of static measurements was between 7.8% and 20.3%, and for latex-free ligatures, the force degradation was between 29.6% and 40.1% when the elastomers were stretched to a distance of 40 mm. In subsequent studies, similar percentages of force degradation were measured, although the experimental conditions or the ligatures were not identical. Oliveria et al. examined latex and latex-free elastics from four manufacturers, wherein the elastomers were stretched to a distance of 20 mm. The force degradation was between 9.8% and 20.1% for the latex elastics and 22% and 28.6% for the latex-free elastomers during the first 24 h of static measurement. Less degradation in strength may be due to less elongation [34]. In a study conducted by Klabunde et al., American Orthodontics’ latex and non-latex elastics were stretched 19.05 mm, whereby the force degradation was approximately 29% after 12 h and 32% after 24 h static stretching in case of the latex elastics, and approximately 48% after 12 h and 56% after 24 h for the non-latex elastics [15]. Kersey et al. used ligatures from the same manufacturer and utilized the same distance as Klabunde, but they measured 17.3% force reduction for the latex and 31.7% force reduction for the non-latex ligatures after 24 h static stretching. These values are closer to the results of this study, but only latex ligatures were measured from the AO manufacturer in this research. [15,32].

During the static test, only one ligature was torn, which was the latex-free Horse carriage^7^, when the elastomers were stretched to a distance of 40 mm.

In the dynamic tests, the latex-containing Gorilla (AO) ligature was the only one that did not break during the test. In case of the worst performing latex-containing elastomer (Parrot (Ormco)), the last elastomer broke at 3.58 h during the 24 h measurement period. Among the latex-free ligatures the Train^8^ (Dentaurum) was the most resistant, but even that only lasted for 12.33 h of cyclic fatigue. Interestingly, the worst performing latex-free ligature, Airplane^12^—which broke after 4.22 h—was more resistant than the worst performing latex ligature. Overall, the static and dynamic tests show that dynamic use damages ligatures more than static use, except for one type of ligature, Impala (Ormco), which did not show significant difference in the results of static and dynamic tests. The results of our tests confirmed our null hypothesis, in which the latex-free elastics suffered a more significant reduction in strength during the cyclic test than the latex-containing elastics. Similar conclusions were reached by Kersey et al. and Liu et al. in their study on the effects of a dynamic environment. Their results show that repeated stretching causes greater force degradation than static stretching, interestingly, such high rates of ligature rupture were not reported [32,35]. This should be emphasized because when an intermaxillary elastic is used to improve occlusion, the elastomers are subjected to repeated stretching while the patient talks, chews and yawns.

In the cyclical examination, significant differences were more often found at 15 mm length than at 40 mm length. This may be because the polymer molecules need more time to regenerate. In the future, it would be interesting to investigate how much time this phenomenon could take or whether it is irreversible and influences the wear time of ligatures. Unfortunately, it cannot be compared with previous studies because they only measured the maximum forces.

The Gorilla ligatures were the only ones that did not fracture during the dynamic test, and only small cracks could be observed with SEM. For the other latex-containing elastomers, fatigue was clearly observable, mainly in the form of well-defined, irregular shaped cracks. In addition, in some cases fluffing was also observed around the hook, which was used to stretch the elastomers. The appearance of fatigue was probably influenced by the composition of the elastomer. However, on the latex-free materials, fatigue was not detectable during the SEM test, but perpendicular and straight fractures were observed without other degradations.

Raman spectroscopy confirmed that there is no significant difference between the composing materials of the latex ligatures, as could be assumed from the percentage of torn ligatures.

A limitation of this study is the in vitro design itself, which does not allow full reproduction of actual clinical conditions, such as the removal and insertion of gingiva after meals or oral hygiene maneuvers. Further clinical trials are recommended to investigate these parameters as well.

## 5. Conclusions

Detailed information about the force degradation characteristics is important for both clinicians and patients. Recommending and selecting the most appropriate type of elastomer gives the patient the opportunity to receive the most effective treatment. The present study suggests that latex ligatures had more balanced power then the latex-free ligatures; therefore, their use may lead to faster results if the patient does not have latex sensitivity. Regardless of the brand and type of the elastics, force degradation is faster by cyclic load. However, latex-free ligatures have higher rates of force degradation; therefore, they should be replaced more frequently. Furthermore, those types of elastomers that performed well in cyclic fatigue tests are more suitable for daytime use, while less resistant types are recommended for night-time wear.

## Figures and Tables

**Table 1:** Main parameters of the tested ligatures according to the technical data sheet.

	Manufacturer/Products	Material	Type	Pulling Force	Inner ø[mm]	ReferenceNumber
1	Ortho/Smile safari	Latex	Lion	Medium	6.4	LOT 990605
2			Zebra	Medium	4.8	LOT 990572
3	Leone	Latex	Red cat	Medium	6.4	K0908-04
4			Green cat	Medium	4.8	K0907-04
5	AO	Latex	Panda	Medium	7.9	LOT 079434
6			Gorilla	Medium	4.8	REF 000-111C
7			Tortoise	Heavy	4.8	LOT 011137
8	Ormco	Latex	Parrot	Light	7.8	LOT 012013216
9			Impala	Extra heavy	4.8	LOT 121971796
10	Dentaurum	Latex free	Horse carrige^7^	Medium	4.8	REF 772-314-01
11			Train^8^	Medium	6.4	REF 772-316-01
12			Airplane^12^	Strong	4.8	REF 772-324-01
13			Space shuttle^13^	Strong	6.4	REF 772-325-00
14	RMO	Latex	Canoe	Heavy	4.8	REF J01121

^7,8,12,13^ The manufacturer does not have an official name for the illustration on the elastomer packaging but can be identified by the numbers in the upper index of the illustrations. These numbers indicate this.

**Table 2:** The results of tensile tests, where *F*_max_ [cN] is the maximum force, SD the standard deviation, *E*_m_ [mm] is the elongation at break in mm and *E*_m_ [%] is the elongation at break in %.

No.	Type	*F*_max_[cN]	SD[cN]	*E*_m_[mm]	SD [mm]	*E*_m_[%]	SD[%]
1	Lion	4667	636	177	1.32	879.7	28.9
2	Zebra	4680	141	136	2.25	904.6	14.9
3	Red cat	3228	238	177	3.28	882.1	16.3
4	Green cat	2801	400	132	2.73	875.6	18.1
5	Panda	3787	254	206	5.18	830.0	20.9
6	Gorilla	3265	608	130	3.83	858.7	25.4
7	Tortoise	3487	209	128	1.68	845.7	11.1
8	Parrot	1596	107	204	5.06	822.6	20.4
9	Impala	4078	203	128	1.32	845.8	8.8
10	Horse carriage^7^	3373	152	219	3.49	1 450	23.1
11	Train^8^	2930	122	252	3.73	1 252	18.5
12	Airplane^12^	3349	268	199	4.90	1 322	32.5
13	Space shuttle^13^	3466	177	228	5.19	1 133	25.8
14	Canoe	2873	272	134	3.04	887.6	20.2

^7,8,12,13^ The name of the elastomers.

**Table 3:** The cyclic fatigue tests’ Tukey statistics in case of Gorilla and Parrot ligatures. The orange color indicates the significant differences with 95% confidence interval. If the change is less than 5%, it is indicated in green.

Cyclic Tensile Fatigue Test								
	6. Gorilla				8. Parrot			
	40 mm		15 mm		40 mm		15 mm	
	Mean Diff. [cN]	p	Mean Diff. [cN]	p	Mean Diff. [cN]	p	Mean Diff. [cN]	p
1 h 0 h	−11.3	0.91	−5.78	0.95	−12.92	0.034	−9.098	0.27
3 h 0 h	−22.22	0.37	−16.20	0.18	−7.98667	0.295	−1.08	0.98
3 h 1 h	−10.92	0.92	−10.42	0.63	4.93333	0.61	8.02	0.45
6 h 0 h	−30.32	0.1	−21.44	0.037	---	---	---	---
6 h 1 h	−19.02	0.53	−15.66	0.21	---	---	---	---
6 h 3 h	−8.1	0.98	−5.24	0.97	---	---	---	---
12 h 0 h	−29.94	0.11	−21.44	0.037	---	---	---	---
12 h 1 h	−18.64	0.56	−15.66	0.21	---	---	---	---
12 h 3 h	−7.72	0.98	−5.24	0.97	---	---	---	---
12 h 6 h	0.38	1	0.00	1	---	---	---	---
24 h 0 h	−47.22	0.003	−29.90	0.002	---	---	---	---
24 h 1 h	−35.92	0.036	−24.12	0.015	---	---	---	---
24 h 3 h	−25	0.25	−13.70	0.34	---	---	---	---
24 h 6 h	−16.9	0.65	−8.46	0.79	---	---	---	---
24 h 12 h	−17.28	0.63	−8.46	0.79	---	---	---	---

**Table 4:** The cyclic fatigue tests’ Tukey statistics in the case of Train^8^ and Airplane^12^ ligatures. There was only one intact ligature at the 12 h; therefore, the statistic was calculated to 6 h. The orange color indicates the significant difference with 95% confidence interval.

Cyclic Tensile Fatigue Test								
	11. Train^8^				12. Airplane^12^			
	40 mm		15 mm		40 mm		15 mm	
	Mean Diff. [cN]	p	Mean Diff. [cN]	p	Mean Diff. [cN]	p	Mean Diff. [cN]	p
1 h 0 h	−16.14	7.54 × 10^−6^	−13.64	0.043	−27.88	0.26	−16.56	0.16
3 h 0 h	−30.14	0	−26.66	1.61 × 10^−4^	−53.46	0.021	−34.08	0.0043
3 h 1 h	−14.0	4.27 × 10^−5^	−13.02	0.055	−25.58	0.32	−17.52	0.14
6 h 0 h	−39.72	0	−35.48	5.42 × 10^−6^	---	---	---	---
6 h 1 h	−23.58	0	−21.84	1.23 x 10^−3^	---	---	---	---
6 h 3 h	−9.58	0.002	−8.82	0.27	---	---	---	---

**Table 5:** The results of the cyclic fatigue and force relaxation were compared by a two-sample t-test. Orange color refers to the significant difference with 95% confidence interval and green color indicates changes less than 5%.

Comparison of the Cyclic Fatigue and Force Relaxation Test												
	40 mm Length						15 mm Length					
	0 h		12 h		24 h		0 h		12 h		24 h	
	Mean Diff. (cN)	p	Mean Diff. (cN)	p	Mean Diff. (cN)	p	Mean Diff. (cN)	p	Mean Diff. (cN)	p	Mean Diff. (cN)	p
1. Lion	2.42	0.82	−10.35	0.46	---	---	−11.20	0.04	−21.18	0.01	---	---
2. Zebra	5.44	0.64	−11.40	0.36	−45.40	0.01	−25.36	0.001	−60.89	4.17 × 10^−4^	−65.85	8.32 × 10^−4^
3. Red cat	7.06	0.67	−25.30	0.15	1.90	0.91	−12.34	0.04	−21.24	0.03	−31.84	9.24 × 10^−5^
4. Green cat	0.92	0.96	15.32	0.45	31.00	0.23	−26.72	0.009	−24.86	0.01	−11.89	0.33
5. Panda	−5.68	0.31	---	---	---	---		0.61	---	---	---	---
6. Gorilla	25.72	0.19	21.58	0.09	15.70	0.21	−11.46	0.67	−24.50	0.06	−22.16	0.33
7. Tortoise	5.48	0.66	---	---	---	---	−48.20	3.80 × 10^−4^	---	---	---	---
8. Parrot	26.02	0.001	---	---	---	---	5.42	0.12	---	---	---	---
9. Impala	1.06	0.96	19.56	0.29	0.08	1.00	−44.90	2.79 × 10^−04^	−36.48	0.002	−43.77	0.01
10. Horse carriage^7^	−1.44	0.86	---	---	---	---	−43.60	9.02 × 10^−5^	---	---	---	---
11. Train^8^	7.82	0.63	---	---	---	---	−36.20	2.90 × 10^−4^	---	---	---	---
12. Airplane^12^	16.50	0.35	---	---	---	---	−72.46	4.23 × 10^−5^	---	---	---	---
13. Space shuttle^13^	21.24	7.32 × 10^−4^	---	---	---	---	−66.32	4.66 × 10^−8^	---	---	---	---
14. Canoe	27.42	0.01	21.68	0.03	8.95	0.43	−30.56	4.59 × 10^−5^	−18.12	0.002	−24.69	0.003
	Latex-free ligatures											
	Latex ligatures											

## Data Availability

The data presented in this study are openly available at All the data presented in this study are openly available at Mendeley Data: Maroti, Peter; Gurdan, Zsuzsanna; Lorinc, Laura; Szabo, Peter; Karadi, Kristof; Told, Roland; Kardos, Kinga; Lukacs, Andras; Turzo, Kinga (2022), “Dataset and supplementary files for the publication entitled “Mechanical characterization and structural analysis of latex-containing and latex-free intermaxillary orthodontic elastics”, Mendeley Data, V2, doi: 10.17632/b2wcpmz9cf.2.

## Supplementary Materials

The following supporting information can be downloaded at: https://www.mdpi.com/article/10.3390/polym14214488/s1. All the data presented in this study are openly available at Mendeley Data: Maroti, Peter; Gurdan, Zsuzsanna; Lorinc, Laura; Szabo, Peter; Karadi, Kristof; Told, Roland; and Kardos, Kinga (2022), “Dataset and supplementary files for the publication entitled “Mechanical characterization and structural analysis of latex-containing and latex-free dental elastomers”, Mendeley Data, V1, doi: 10.17632/b2wcpmz9cf.1.
