# Supplementary figures and images for: Mechanical Characterization and Structural Analysis of Latex-Containing and Latex-Free Intermaxillary Orthodontic Elastics
Source: Polymers (Basel). 2022 Oct 23;14(21):4488. doi: 10.3390/polym14214488 (PMC9654203; doi:10.3390/polym14214488)

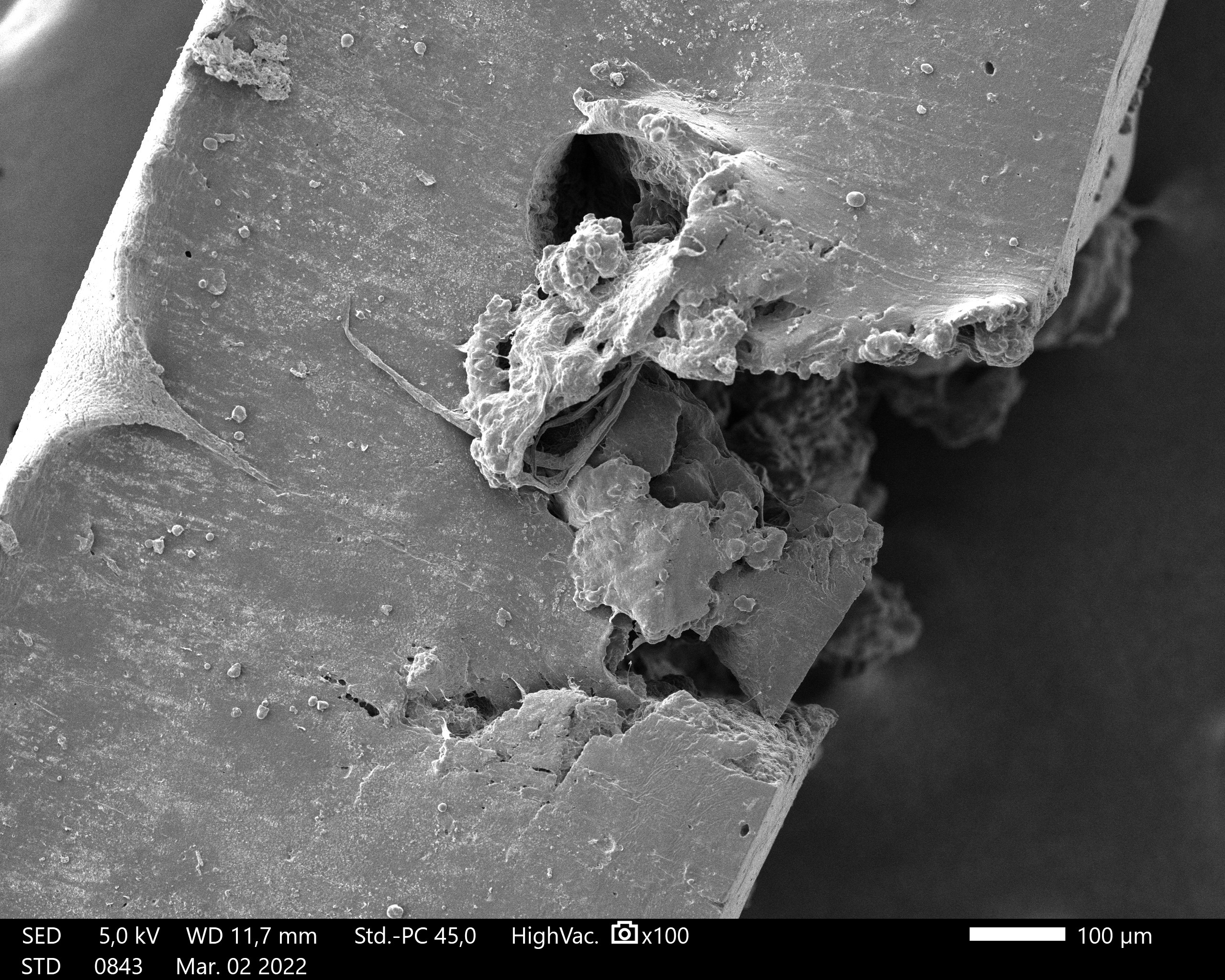

Supplement: Supplementary file 1 [file polymers-14-04488-s001.zip › SEM/01 - Lion 100x.jpg]

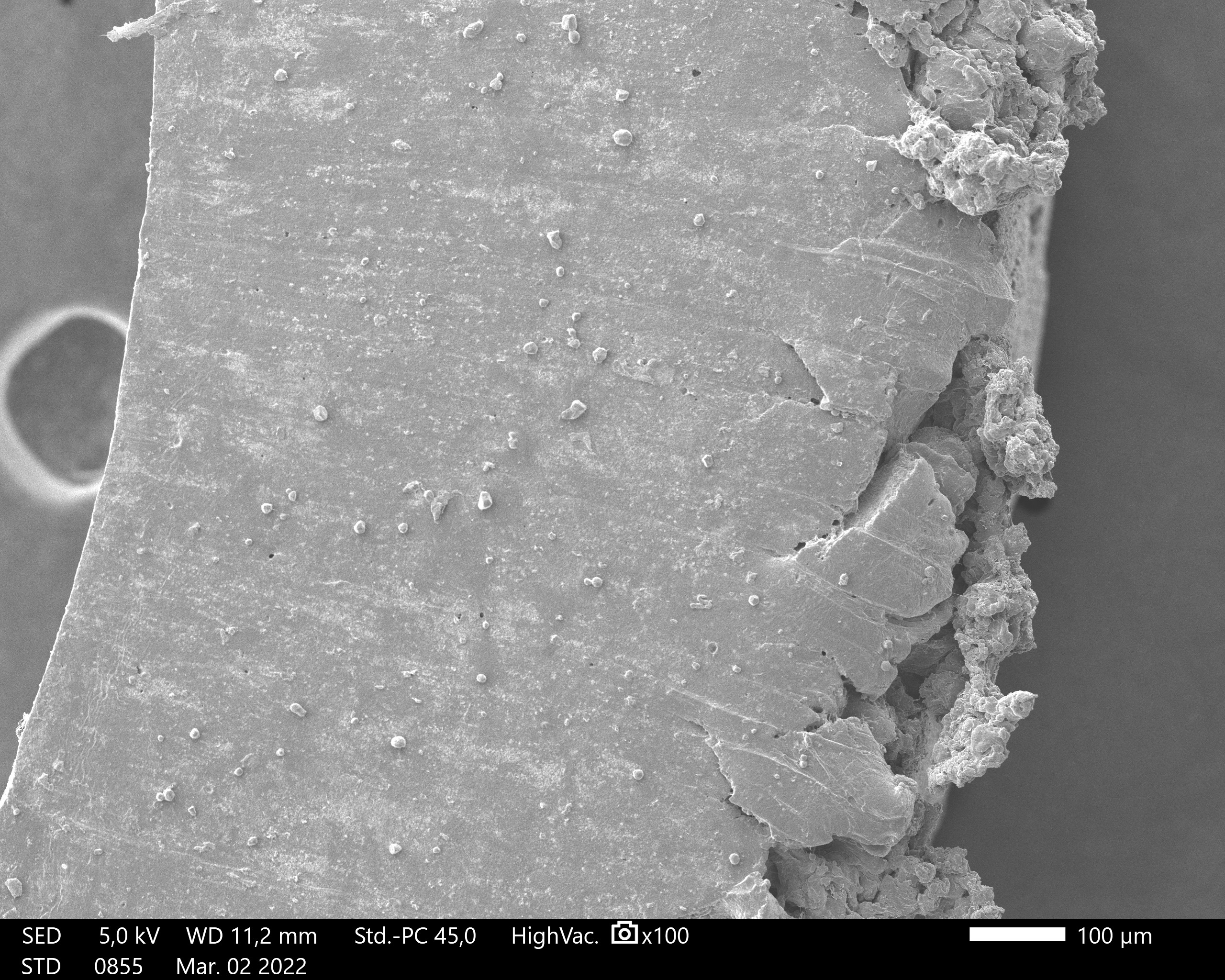

Supplement: Supplementary file 1 [file polymers-14-04488-s001.zip › SEM/02 - Zebra 100x.jpg]

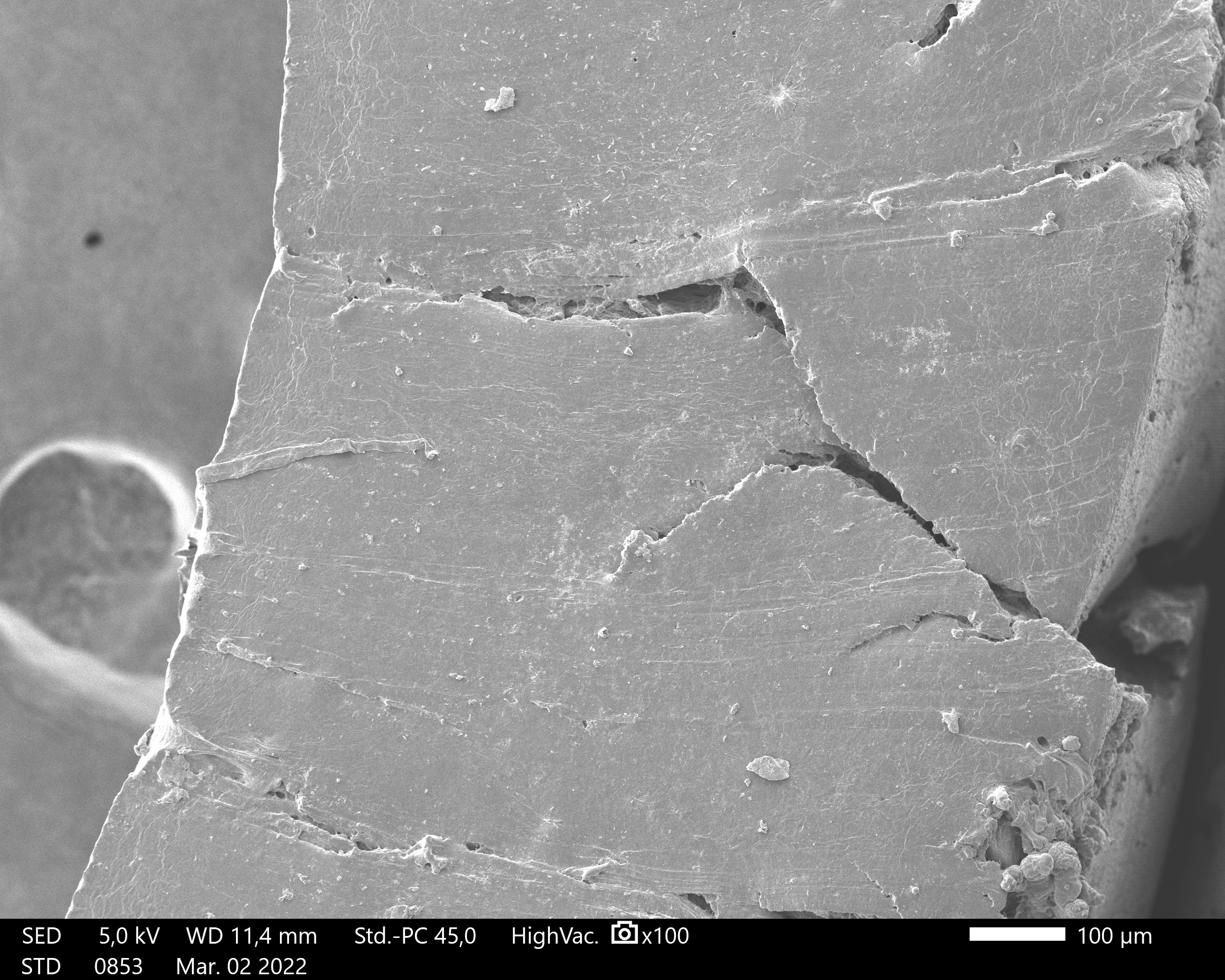

Supplement: Supplementary file 1 [file polymers-14-04488-s001.zip › SEM/03 - Red cat 100x.jpg]

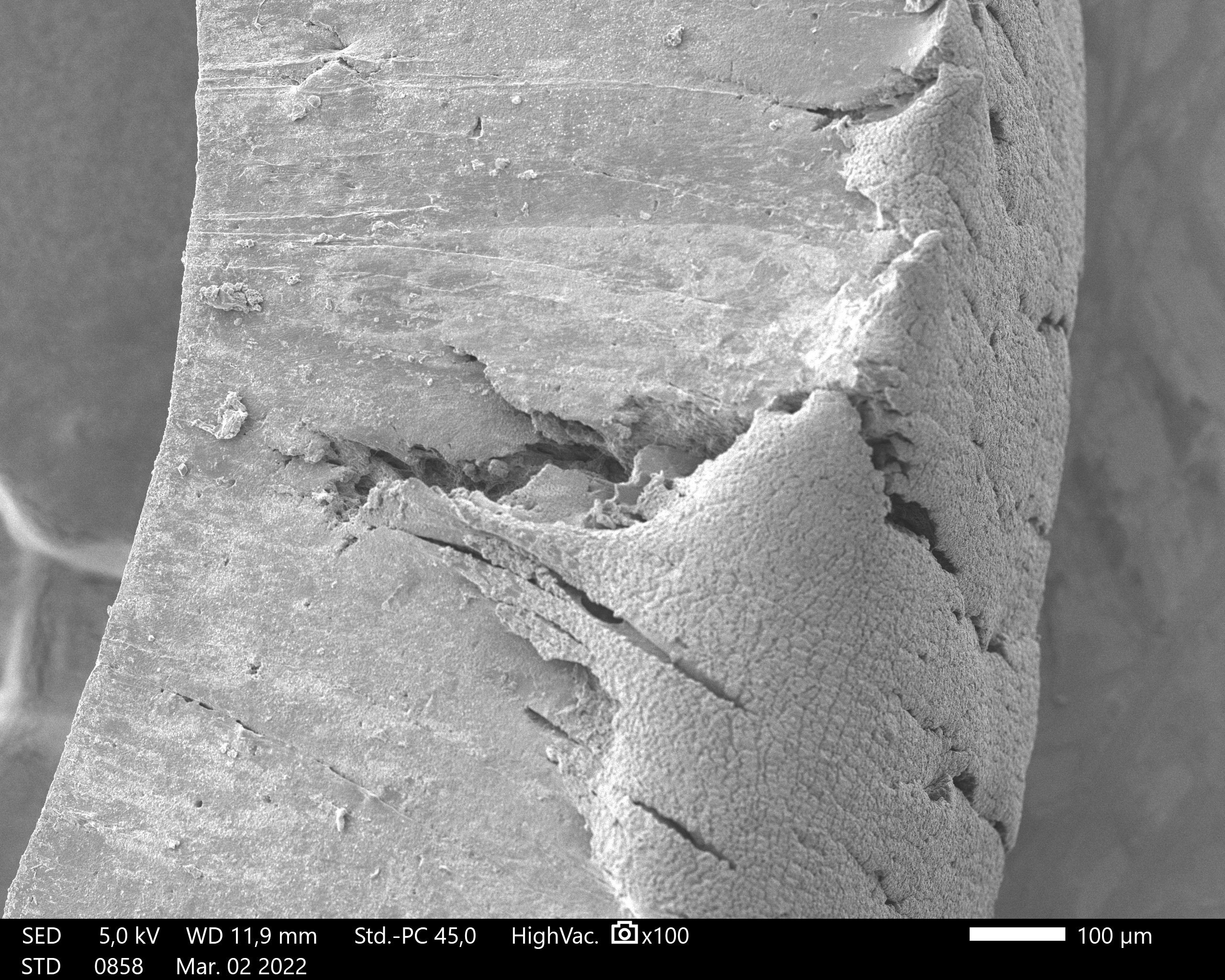

Supplement: Supplementary file 1 [file polymers-14-04488-s001.zip › SEM/04 - Green cat 100x.jpg]

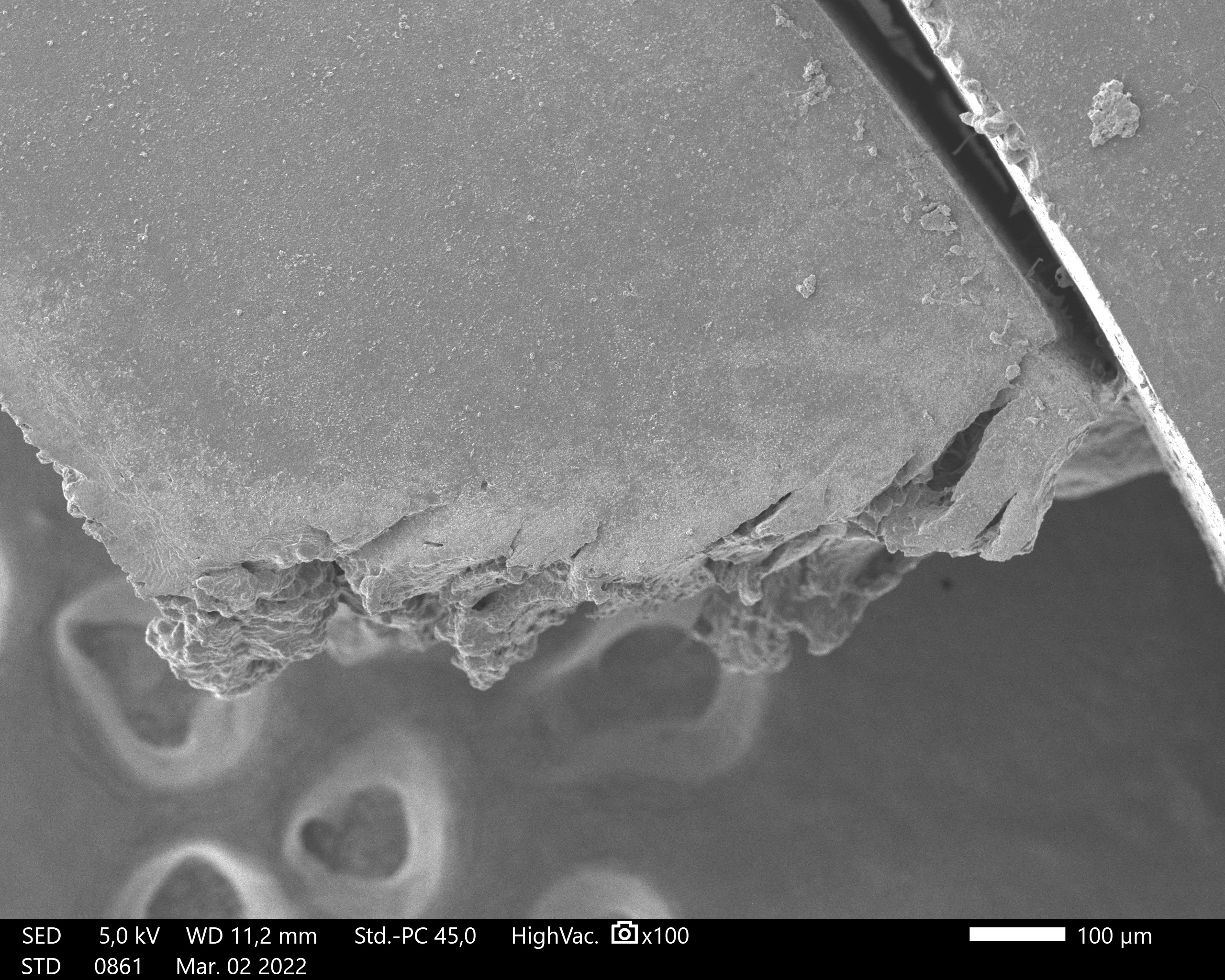

Supplement: Supplementary file 1 [file polymers-14-04488-s001.zip › SEM/05 - Panda 100x.jpg]

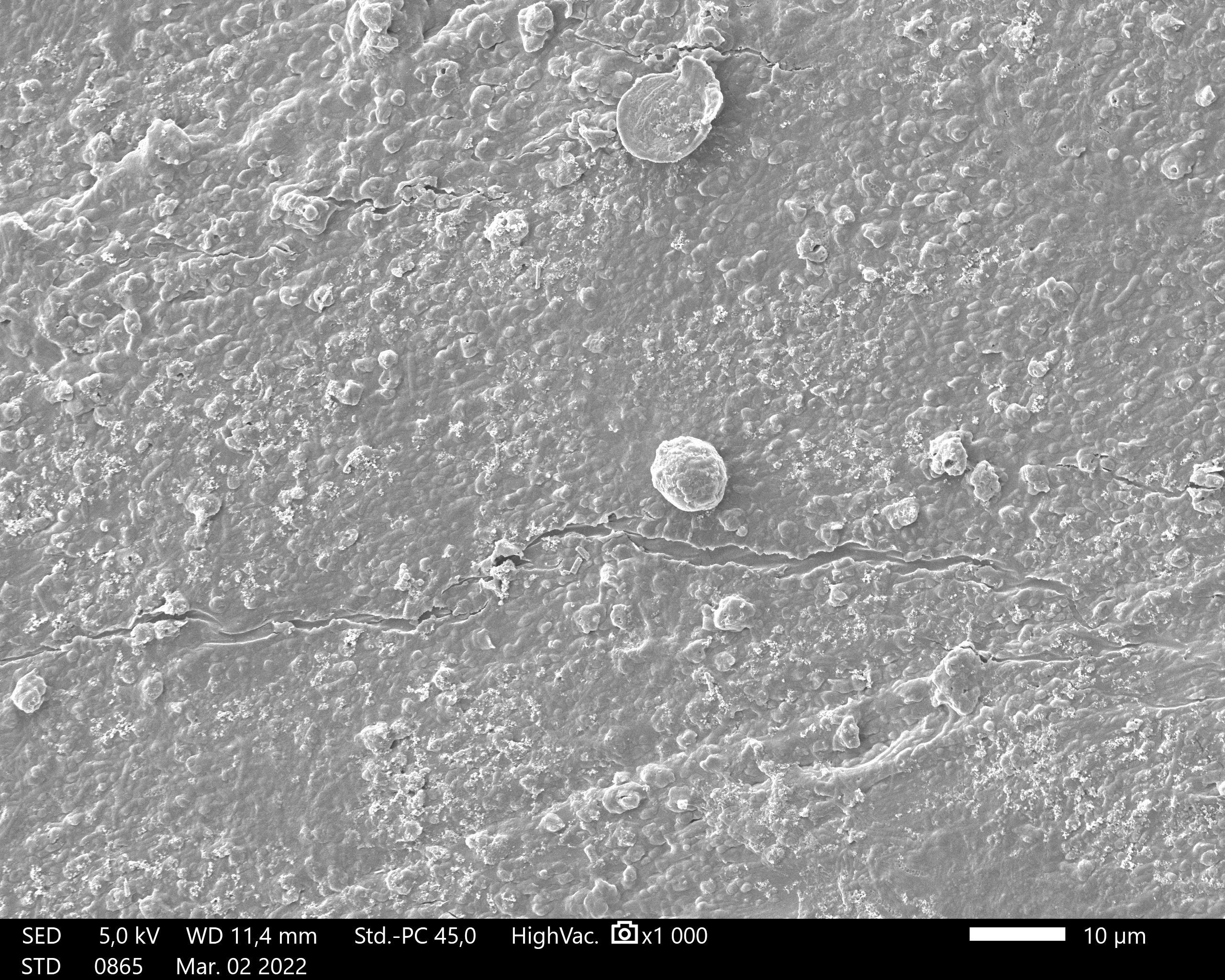

Supplement: Supplementary file 1 [file polymers-14-04488-s001.zip › SEM/06 - Gorilla 1000x.jpg]

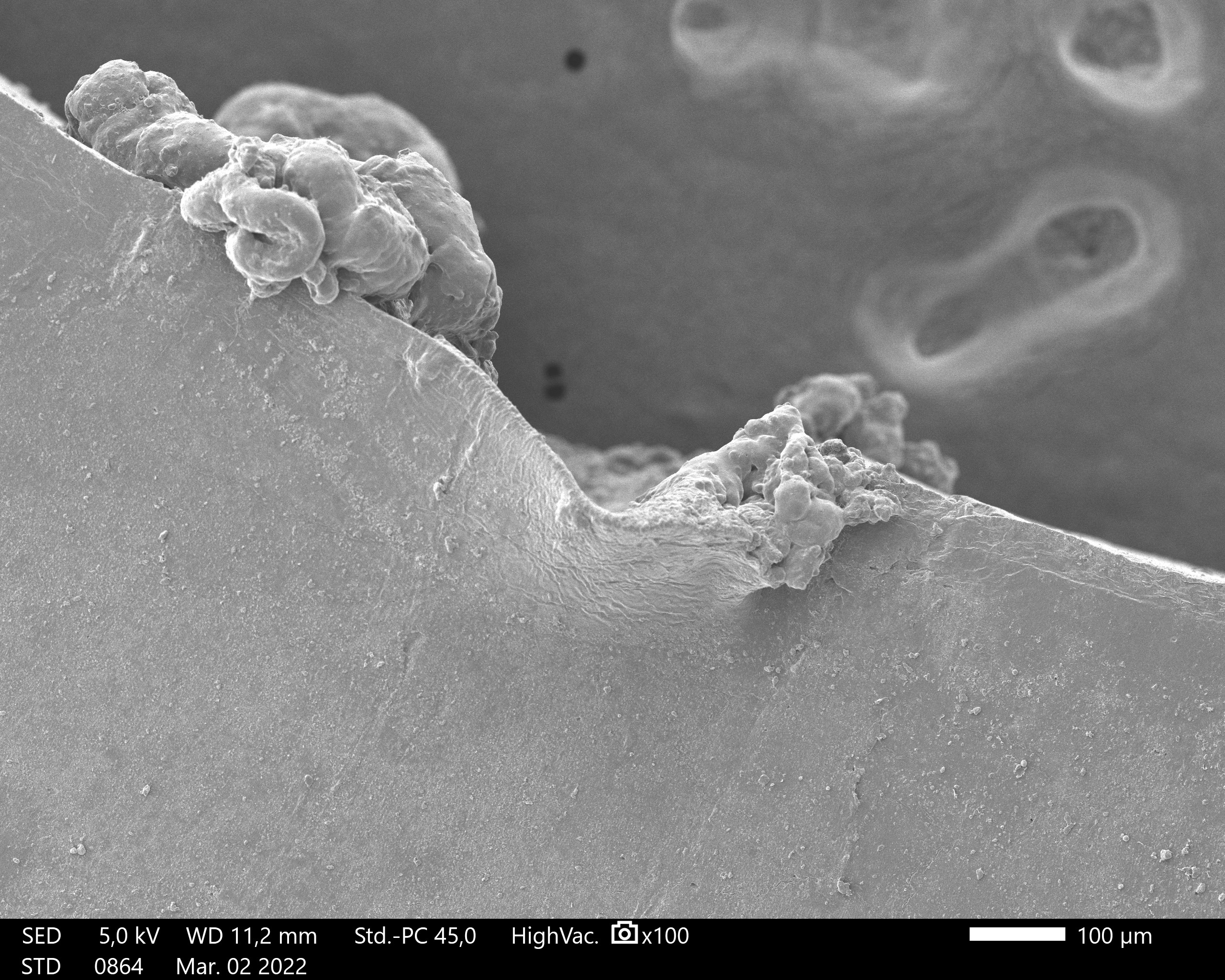

Supplement: Supplementary file 1 [file polymers-14-04488-s001.zip › SEM/06 - Gorilla 100x.jpg]

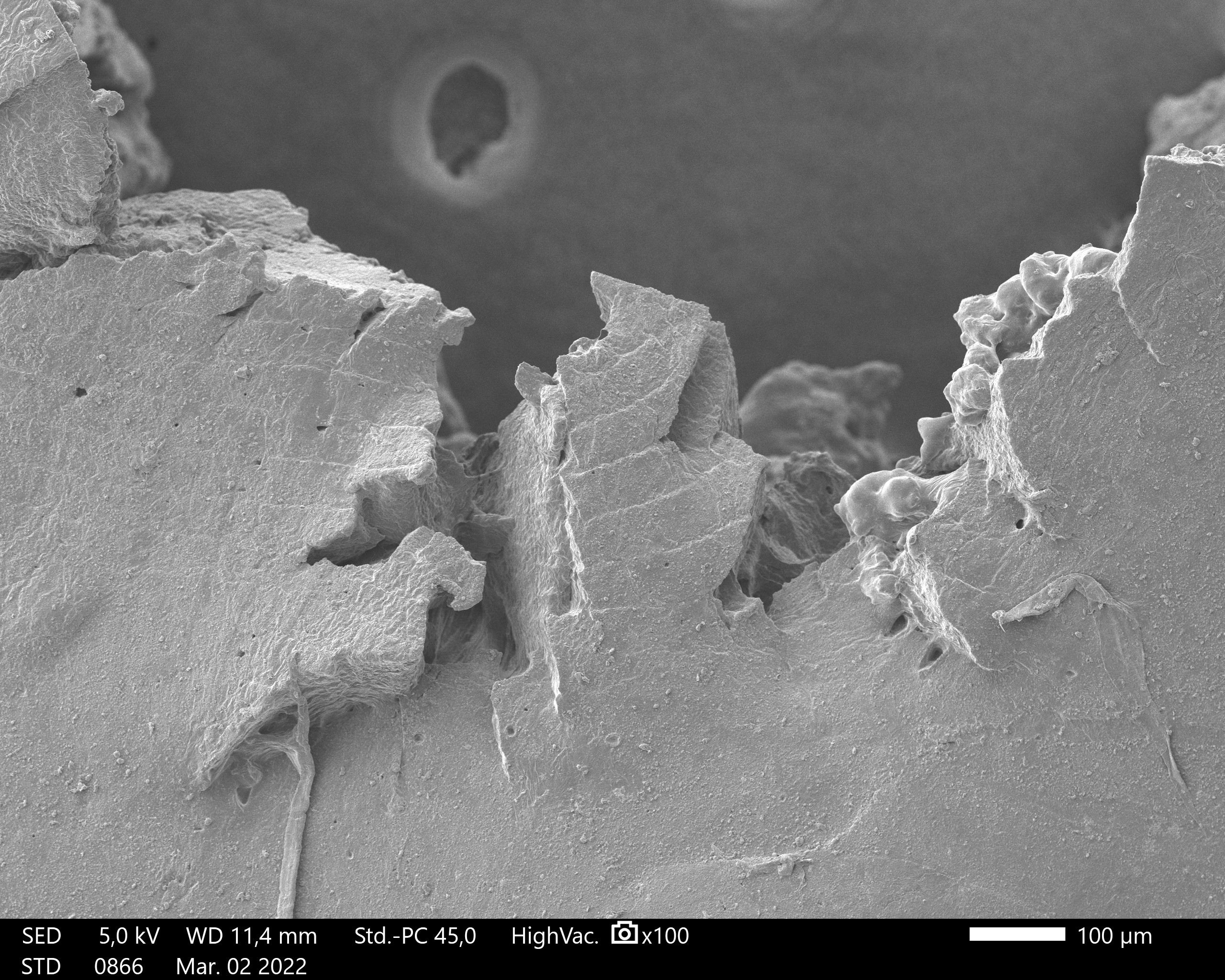

Supplement: Supplementary file 1 [file polymers-14-04488-s001.zip › SEM/07 - Tortoise 100x.jpg]

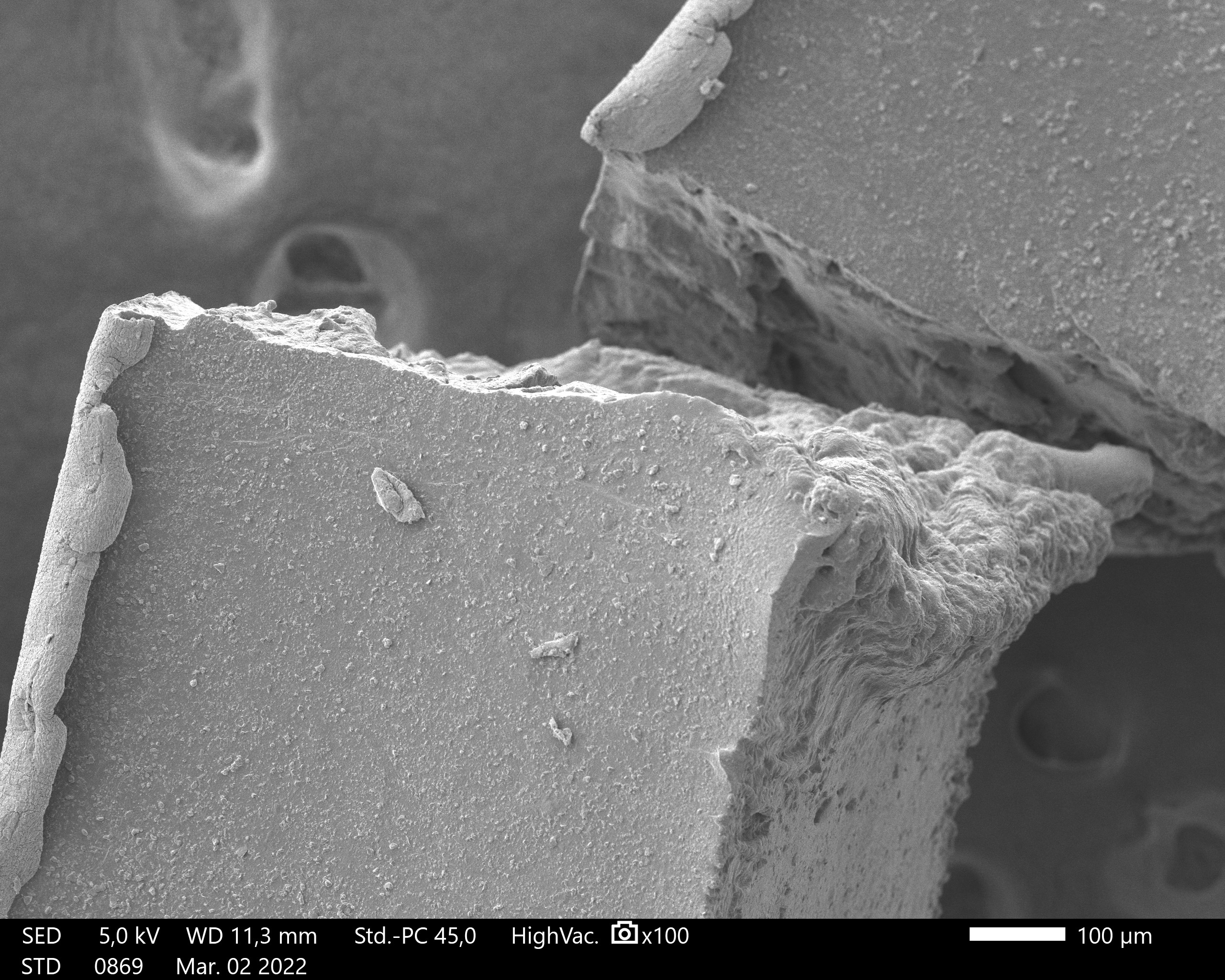

Supplement: Supplementary file 1 [file polymers-14-04488-s001.zip › SEM/08 - Parrot 100x.jpg]

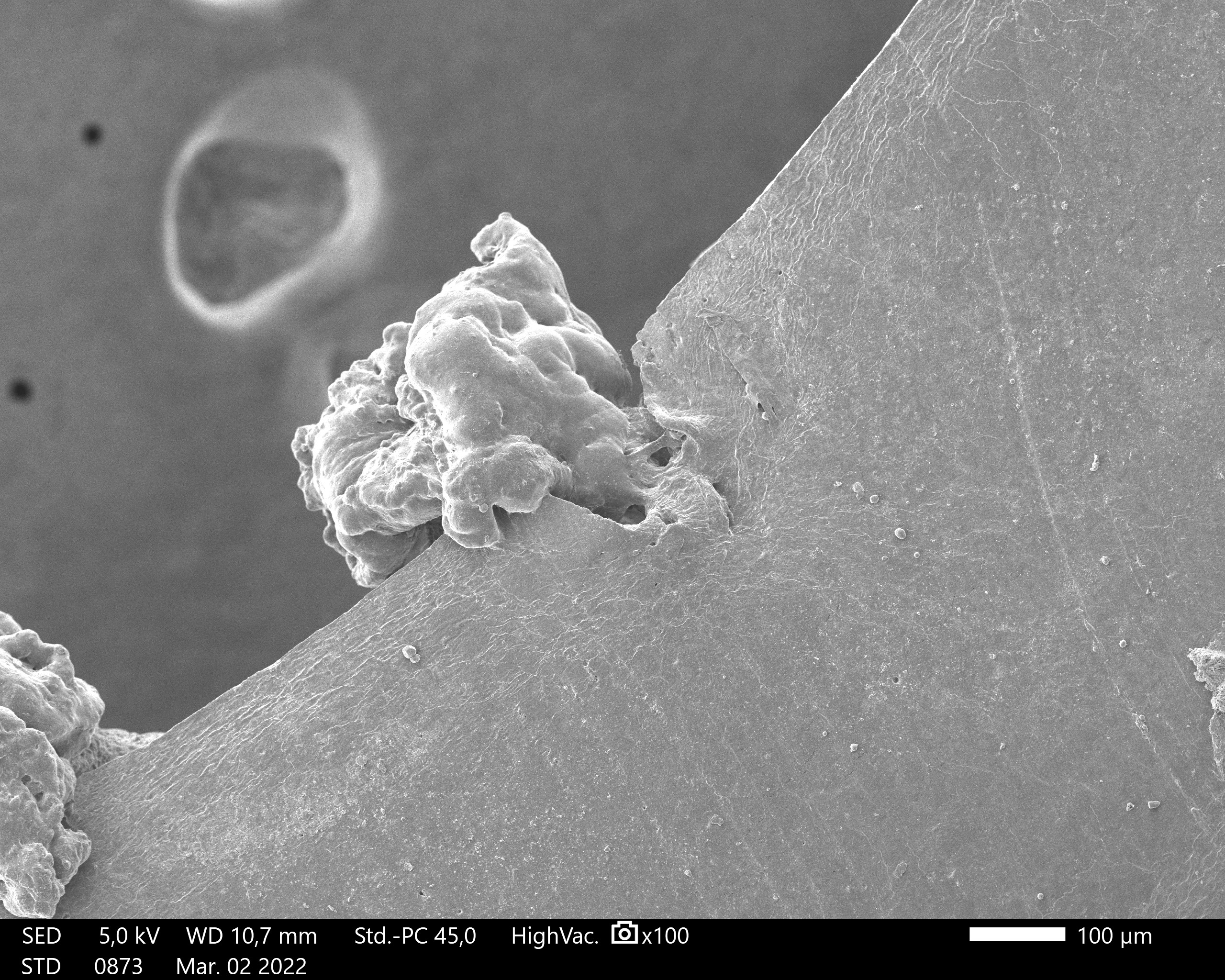

Supplement: Supplementary file 1 [file polymers-14-04488-s001.zip › SEM/09 - Impala 100x.jpg]

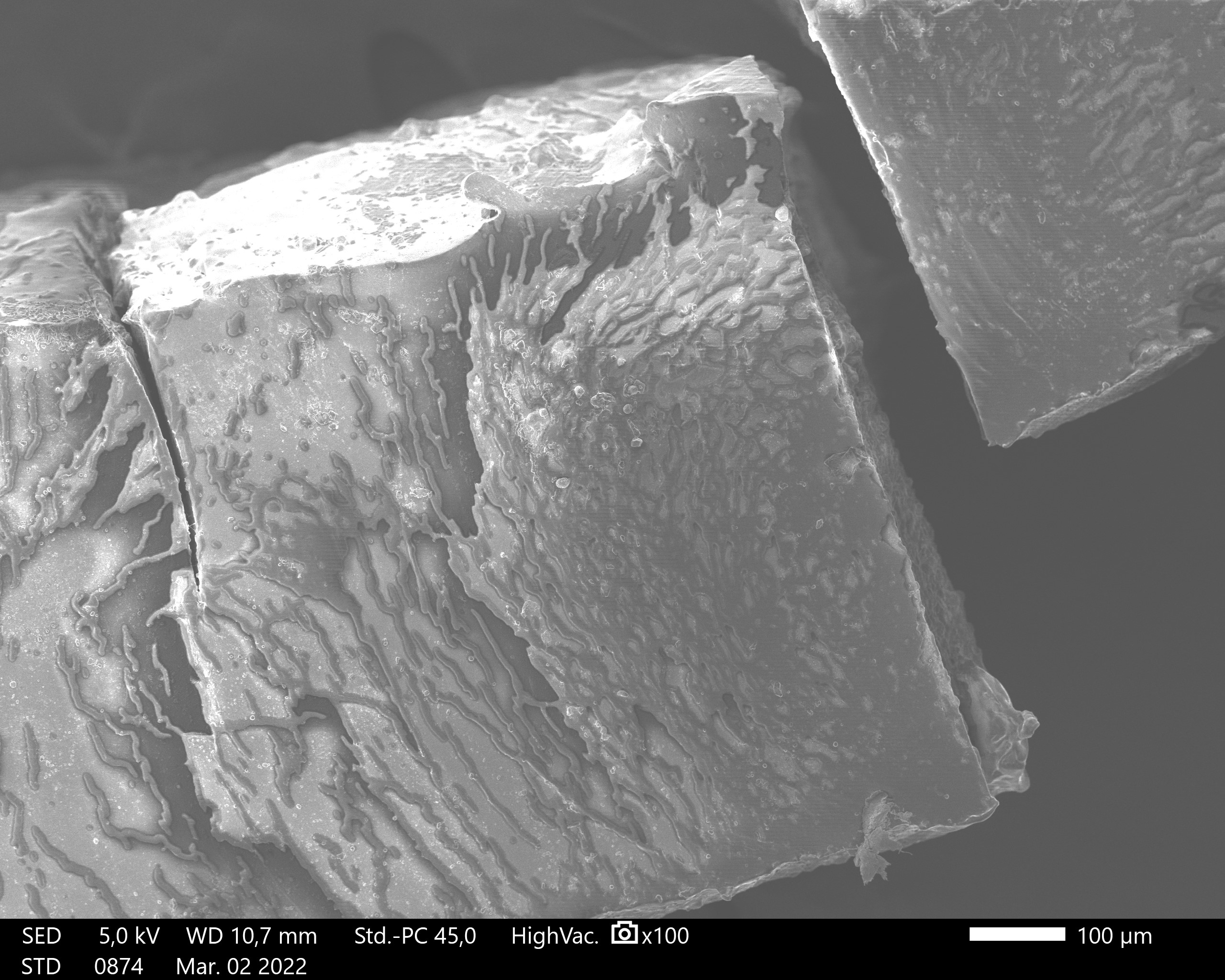

Supplement: Supplementary file 1 [file polymers-14-04488-s001.zip › SEM/10 - Horse carriage 100x.jpg]

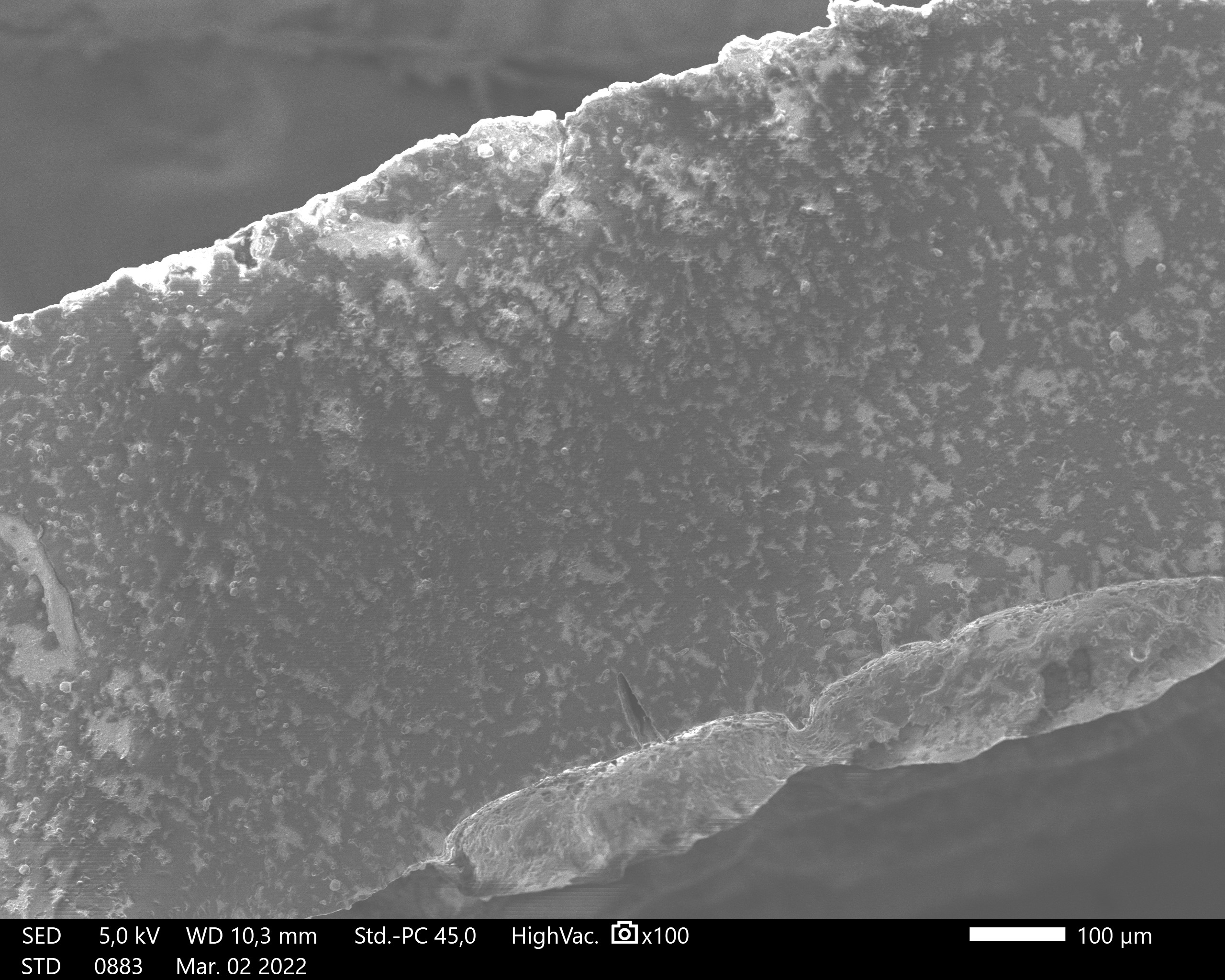

Supplement: Supplementary file 1 [file polymers-14-04488-s001.zip › SEM/11 - Train 100x.jpg]

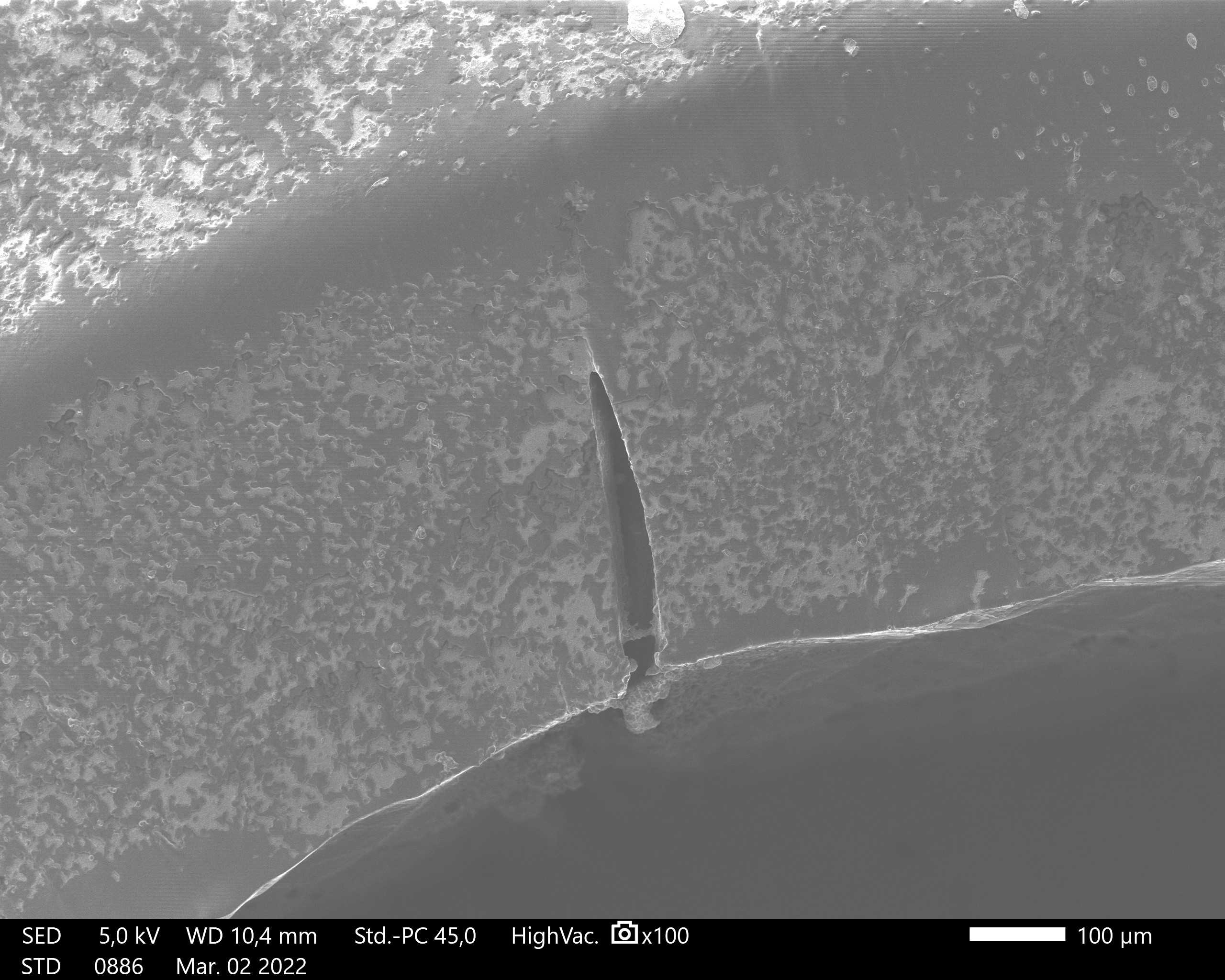

Supplement: Supplementary file 1 [file polymers-14-04488-s001.zip › SEM/12 - Airplane 100x.jpg]

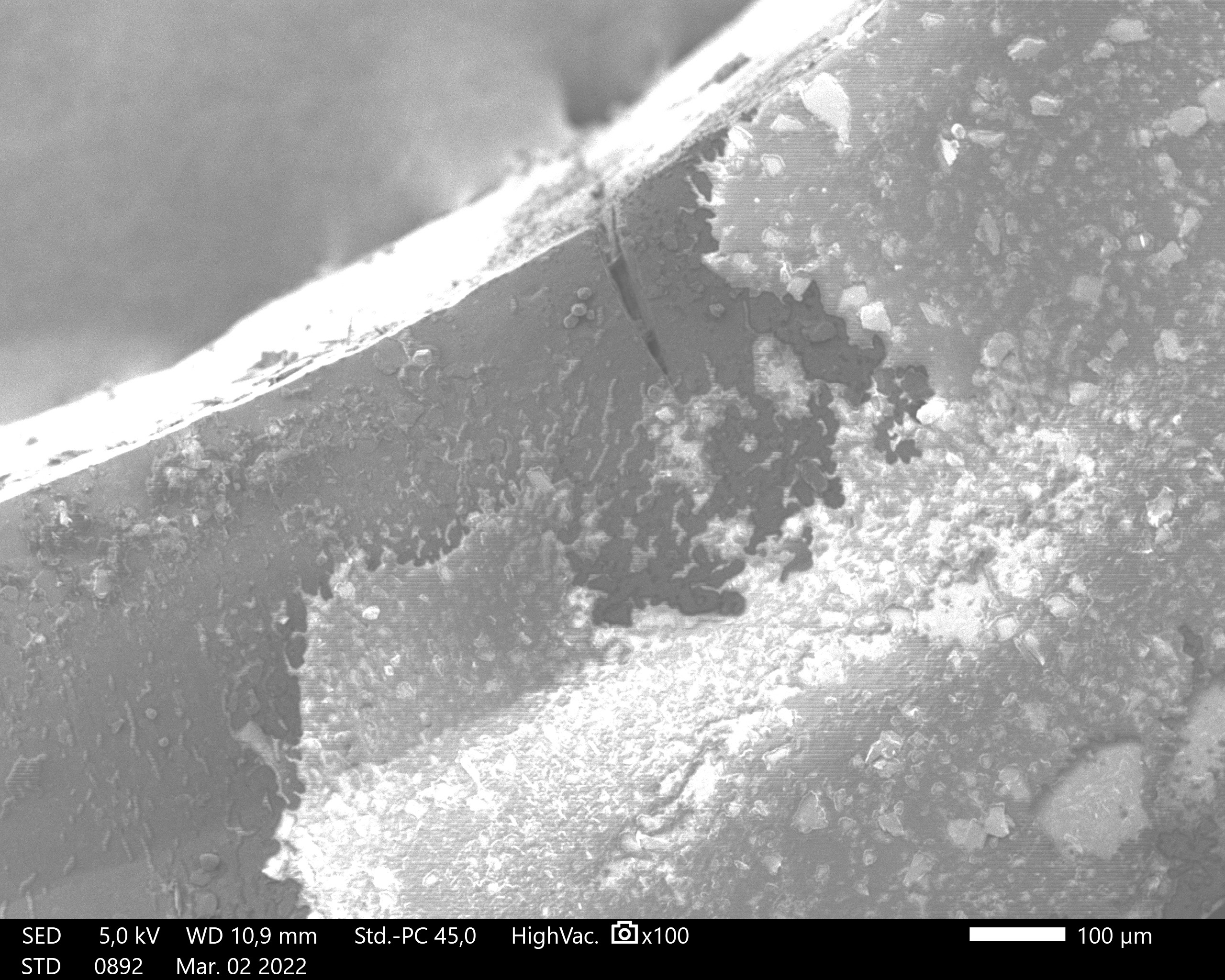

Supplement: Supplementary file 1 [file polymers-14-04488-s001.zip › SEM/13 - Space shuttle 100x.jpg]

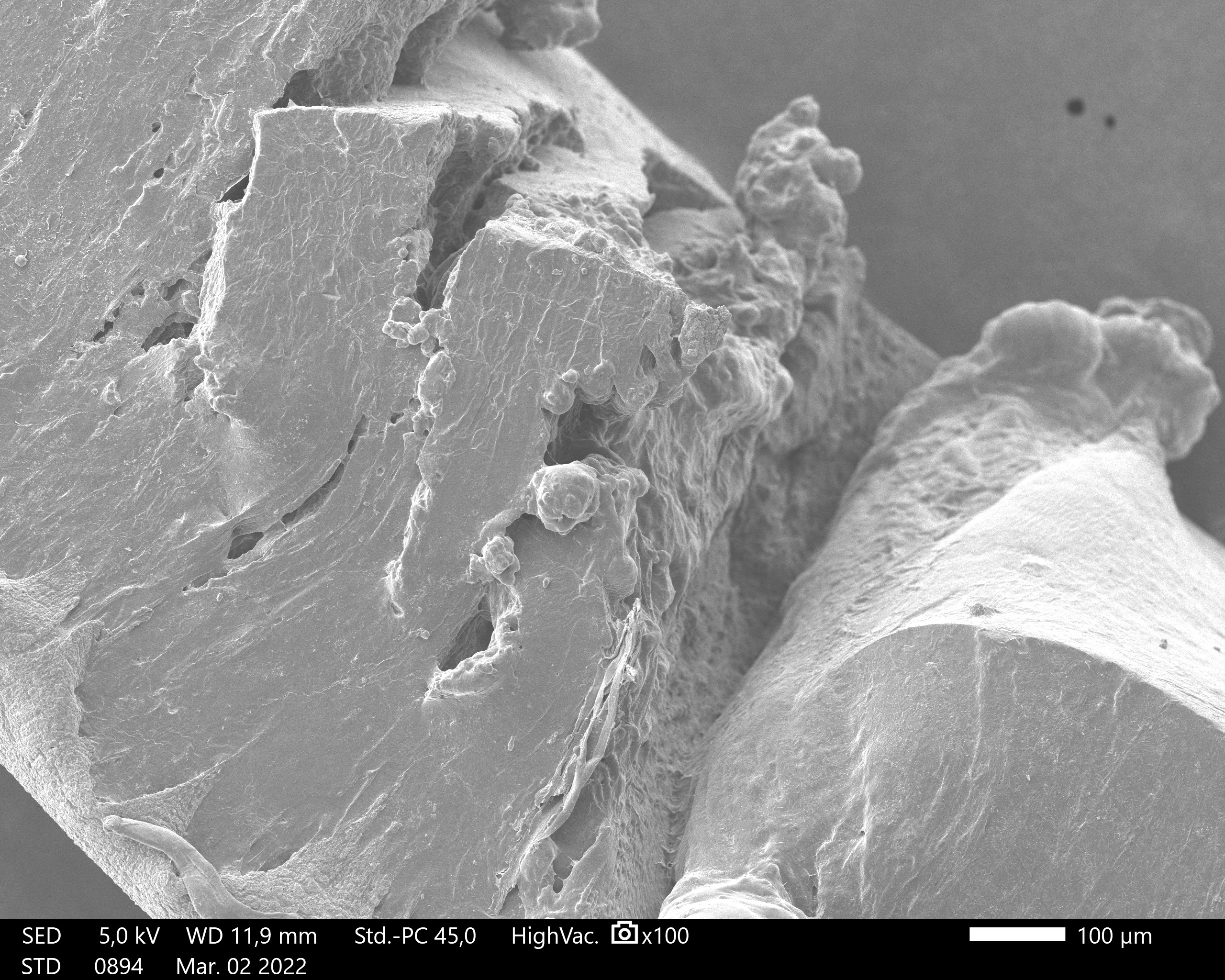

Supplement: Supplementary file 1 [file polymers-14-04488-s001.zip › SEM/14 - Canoe 100x.jpg]
